# Deterministic and Stochastic Study for a Microscopic Angiogenesis Model: Applications to the Lewis Lung Carcinoma

**DOI:** 10.1371/journal.pone.0155553

**Published:** 2016-05-16

**Authors:** Marek Bodnar, Pilar Guerrero, Ruben Perez-Carrasco, Monika J. Piotrowska

**Affiliations:** 1 Institute of Applied Mathematics and Mechanics, Faculty of Mathematics, Informatics and Mechanics, University of Warsaw, Banacha 2, 02-097 Warsaw, Poland; 2 Department of Mathematics, University College London, Gower Street, London WC1E 6BT, United Kingdom; University of Leeds, UNITED KINGDOM

## Abstract

Angiogenesis modelling is an important tool to understand the underlying mechanisms yielding tumour growth. Nevertheless, there is usually a gap between models and experimental data. We propose a model based on the intrinsic microscopic reactions defining the angiogenesis process to link experimental data with previous macroscopic models. The microscopic characterisation can describe the macroscopic behaviour of the tumour, which stability analysis reveals a set of predicted tumour states involving different morphologies. Additionally, the microscopic description also gives a framework to study the intrinsic stochasticity of the reactive system through the resulting Langevin equation. To follow the goal of the paper, we use available experimental information on the Lewis lung carcinoma to infer meaningful parameters for the model that are able to describe the different stages of the tumour growth. Finally we explore the predictive capabilities of the fitted model by showing that fluctuations are determinant for the survival of the tumour during the first week and that available treatments can give raise to new stable tumour dormant states with a reduced vascular network.

## Introduction

Angiogenesis, the formation of new vessels from pre-existing ones, is a normal and vital process in growth and development of animal organisms. It is required during the repair mechanism of damaged tissues like wound healing processes. However, angiogenesis is also essential in the transition of avascular forms of solid tumours into cancers that are able to metastasise and cause the lethal outcome of the disease. Hence, angiogenesis promotes cancer growth.

In general, for tumours reaching size of 1-2 mm^3^ the necrotic core inside the tumour is formed [1, 2]. At this stage, cancer cells start to secrete angiogenic factors *e.g*. FGF, VEGF, VEGFR, Ang1 and Ang2, which promote proliferation and differentiation of endothelial cells, smooth muscle cells, and fibroblasts, initiating the formation of new blood vessels. Those vessels provide the nutrients for growing cancer mass and help to remove the metabolism waste products. On the other hand, achieving a better control on angiogenesis would also improve the cancer treatment allowing anti-cancer drugs to penetrate efficiently the tumour structure through a good functioning vessel network, and hence reduce the tumour mass.

One of the most well known mathematical models describing the influence of angiogenesis processes on the tumour dynamics was proposed by Hahnfeldt *et al*. [3]. This model and its extensions were later studied, among others, by d’Onofrio & Gandolfi [4–6] and by Piotrowska & Foryś [7, 8]. Additionally, within last years, different treatment protocols were introduced to the Hahnfeldt *et al*. type models, and optimal control theory was applied to these modified models to find a proper timing and dosing of drug administration (see e.g. [9–13]), settling those models as the paradigm of angiogenesis modelling. However, global stability of a positive steady state is a weak point of that type of models without delays, since newly formed vessels usually have highly unstable structure. Moreover, feedback loops observed in the biological systems can lead to oscillatory dynamics, [14]. To reflect the complex nature of the vessels formation process, Arakelyan *et al*. [15] proposed an intricate computational model, which was compared with implanted human ovarian carcinoma reported in [16]. Next, that complex model was simplified to the system of three equations with two time delays. The delays presented in this latter model reflect the length of feedback loops considered as detailed reactions in the original computational model [15]. Later in [17], it was showed that independently of the magnitude of delays, the positive steady state is always unstable and the model can not reflect a stable behaviour of newly formed vessels observed for less aggressive tumours, [18]. In [19], combining the ideas presented by Hahnfeldt *et al*. [3] and Agur *et al*. [20] a model of three differential equations with discrete delays describing the process of formation of new vessels that could reflect both, stable and unstable, structures of vessels observed in reality was proposed. Next, in [21] a detailed stability analysis of this model was performed showing that for some parameters a hysteresis loop can be observed and multiple stability switches can occur with increasing time delay.

The models described above are mean-field, in the sense that they deal with the dynamics of the tumour without taking into account its spatial organisation. On the other hand, there are several models studying this spatial dependence focusing on the patterning of the vascular network without delving into the microscopic details [22–25].

In the current work we aim to bring together the microscopic reactions occurring at cellular level with the mean-field models looking for the essential features of the non-linear interaction between cells, vessels and endothelial growth factors, with the goal to build a solid understanding that can be expanded into the spatial models.

Such a microscopic description starts by identifying the system of reactions that governs the tumour growth. The advantage of having the microscopic description is twofold. On one hand, a formal derivation of the macroscopic system of ODE and the meaning of the parameters become available, giving a justification for the type of models considered in [19] and [21].

In contrast, the microscopic description is intrinsically stochastic, formally described by a multivariate birth-death process [26], that can be decisive in the macroscopic tumour dynamics giving place to effects not present in a pure deterministic description [27–29], and feature an upcoming effort to integrate them into experimental data [30, 31]. For this reason we also analyse the stochastic system through exact simulations of the kinetic reactions and by using the corresponding Chemical Langevin Equation [32] that gives analytical insight on the effect of intrinsic noise in the macroscopic evolution of the tumour.

Additionally, to give soundness to the prediction of the model we use experimental data to give meaningful values to each parameter of the model. The result is a working model able to predict the dynamics of the Lewis lung carcinoma.

## Materials and Methods

### Microscopic description

The dynamical evolution of the tumour is determined by the non—linear interactions between the tumour cells, *N*, and the effective number of blood vessel cells, *V*, or to be precise the number of endothelial cells that lines the interior surface of blood vessels. This interaction takes place by means of VEGF, the regulating proteins secreted by the tumour cells that promote the angiogenesis process, *P*. It is also useful to define the relative quantity *E* ≡ *V*/*N*, which measures the number of vessel cells that are available per tumour cell *i.e*. gives a measure of the amount of nutrient each tumour cell receives.

The different events characterising the dynamics of the system take place at a microscopic level as a set of events changing individually the population of each species. Following the molecular analogue, such events will be referred here on as reactions. Some of these reactions depend on the absolute number of tumour cells/vessels/proteins (denoted by *N*/*V*/*P*, respectively) while others depend on the concentration of each of those species in the system $N^{*}\equiv \frac{N}{\Omega }$, $V^{*}\equiv \frac{V}{\Omega }$, $P^{*}\equiv \frac{P}{\Omega }$, where *Ω* is the parameter relating concentrations with the number of cells (or molecules), giving a measure of the size of the system. The larger is *Ω* for a certain concentration, the greater will be the corresponding number of cells (proteins). The parameters used to describe the particular reactions are summarised in Table 1.

**Table 1:** Microscopic parameters.

**Cell Parameters**	
*α*	maximal proliferation rate
*K*	characteristic tumour size
*b*_1_	maximal nutrient supply
*c*_1_	switching value of *E* for nutrient shortage
*δ*_*N*_	maximal death rate
*n*, *n*_1_, *n*_2_	Hill exponents
**Protein Parameters**	
*a*_2_	maximal protein production rate
*c*_2_	switching value of *E* for protein production
*δ*_*P*_	degradation rate
**Vessel Parameters**	
*b*_3_	maximal growth rate
*a*_3_	maximal death rate
*m*_3_	characteristic protein concentration
*n*_3_, *n*_4_	Hill exponents

#### Cell reactions

**Fig 1:**
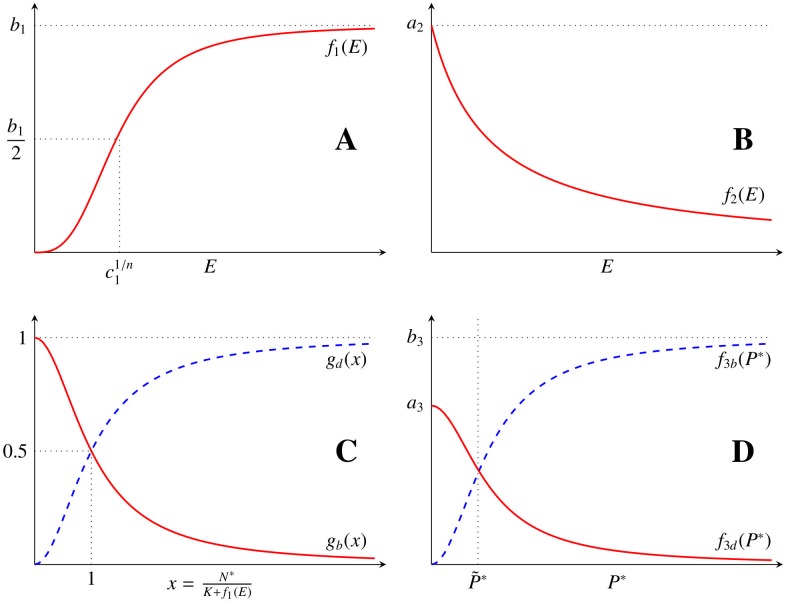
Rate dependences characterizing tumour, vascular and VEGF dynamics. (A) Effective nutrients received *f*_1_(*E*) for *c*_1_ = 1 and *n* = 3. (B) VEGF production rate *f*_2_(*E*), dependence on the effective nutrients available. (C) Rates of proliferation (solid line) and death (dashed line) for the tumour cells with *n*_1_ = 2. (D) Rates of proliferation and death for the vessels dependence with VEGF concentration for *n*_3_ = *n*_4_ = 2.

The first considered reaction is the cell proliferation in which a cell can divide giving place to two daughter cells,

$$\begin{array}{c} N\overset{\alpha g_{b}\left(\frac{N^{*}}{K+f_{1}\left(E\right)}\right)}{\to }N+N \end{array}$$ (1)

where *α* > 0 is the maximal proliferation rate and *g*_*b*_(·) is a decreasing function capturing intracellular competition for resources. Such a competition will increase with tumour cell concentration N*, causing a reduction in the proliferation rate. On the other hand, the proliferation capability increases with the amount of nutrients that the tumour cells receive through the vessels *f*_1_(*E*). These assumptions capture a switching behaviour in which the cells proliferate if the environmental conditions are favourable (N* < *K* + *f*_1_(*E*)) (Fig 1A). Thus, for a large concentration of available nutrients, the cells duplicate with a maximum rate *α*, (*g*_*b*_(0) = 1), while the division decay for a lower concentration of nutrients (lim_*x* → ∞_ *g*_*b*_(*x*) = 0). In particular, we assume that *g*_*b*_(·) has the Hill form

$$\begin{array}{c} g_{b}\left(x\right)=\frac{1}{1+x^{n_{1}}} \end{array}$$ (2)

with *n*_1_ > 0.

The actual concentration threshold (*K* + *f*_1_(*E*)) depends non-linearly on the concentration of endothelial cells through *E* and it is related to the maximal tumour size that can be reached assuming the effective vessel density is equal to *E*. The parameter *K* corresponds to the maximal tumour size if no vasculature is present. Thus, following the ideas of [19–21], it is assumed that *f*_1_(*E*) is an increasing Hill function of *E* such that *f*_1_(0) = 0. Additionally, in a nutrient saturation environment there is a maximum response *b*_1_.

$$\begin{array}{c} f_{1}\left(E\right)=\frac{b_{1}E^{n}}{c_{1}+E^{n}} \end{array}$$ (3)

with *b*_1_, *c*_1_, *n* > 0 (see Fig 1A).

On the other hand, under hostile conditions cells can also die following similar arguments of competition and saturation of resources as the growth rate,

$$\begin{array}{c} N\overset{\delta_{N}g_{d}\left(\frac{N^{*}}{K+f_{1}\left(E\right)}\right)}{\to }\emptyset \end{array}$$ (4)

where *δ*_*N*_ is the maximum death rate and function *g*_*d*_(·) grows with the concentration of tumour cells and decreases with the nutrient supply, and analogously to [19–21], is assumed a Hill increasing function,

$$\begin{array}{c} g_{d}\left(x\right)=\frac{x^{n_{2}}}{1+x^{n_{2}}} \end{array}$$ (5)

(see Fig 1C). Note that this description assumes the same characteristic tumour size *K* + *f*_1_(*E*) for both, the birth and death rate, as a first approximation in order to reduce the complexity of the model.

#### Protein reactions

The regulation proteins, that stimulate vessel growth, are secreted by tumour cells,

$$\begin{array}{c} N\overset{f_{2}\left(E\right)}{\to }N+P. \end{array}$$ (6)

The rate of stimulatory protein secretion *f*_2_(*E*) also depends on the supply of nutrients to the tumour cells (*E*) and it is assumed to be a non-negative decreasing function of *E* that satisfies (lim_*E* → ∞_ *f*_2_(*E*) = 0), see [20]. On the other hand, if no vascular network is present, the VEGF proteins are secreted at a maximum rate *a*_2_ (*f*_2_(0) = *a*_2_) with a characteristic concentration *c*_2_, giving place to the used expression for

$$\begin{array}{c} f_{2}\left(E\right)=\frac{a_{2}c_{2}}{c_{2}+E} \end{array}$$ (7)

(see Fig 1B).

Additionally, the proteins degrade with a constant rate *δ*_*P*_,

$$\begin{array}{c} P\overset{\delta_{P}}{\to }\emptyset . \end{array}$$ (8)

#### Vessel reactions

Finally, the regulatory proteins secreted by the tumour cells control the angiogenesis process, thus

$$V\;\overset{f_{3b}\left(P^{*}\right)}{\to }\;V\;+\;V$$ (9)

$$V\;\overset{f_{3d}\left(P^{*}\right)}{\to }\;\emptyset .$$ (10)

Similarly, to the tumour cell proliferation, the vascular generation/death is controlled by two switching mechanisms that follow a rate proportional to the number of vessel cells and a factor dependent on the protein concentration reaching maximum generation/death rates *b*_3_, *a*_3_ > 0, and, following the ideas of [20], can be described as

$$f_{3b}\left(x\right)\;=\;\frac{b_{3}x^{n_{3}}}{m_{3}^{n_{3}}+x^{n_{3}}}$$ (11)

$$f_{3d}\left(x\right)\;=\;\frac{a_{3}m_{3}^{n_{4}}}{m_{3}^{n_{4}}+x^{n_{4}}}$$ (12)

(see Fig 1B), where *n*_3_, *n*_4_ > 0 and *m*_3_ is a characteristic protein concentration under which the production and degradation of vessels reach half of their maximum values. Specifically, in the case *b*_3_ = *a*_3_, *m*_3_ is the protein concentration for which vessel cell birth and death are in balance. More detailed vessel birth and death reactions (9) and (10) would include competition between vessels for the available protein. This is not included in this model as a first approximation to vessel cell dynamics. Table 1 gathers the introduced parameters for the three species and their interpretation.

### Macroscopic system

Once the reaction scheme is set, a coarse grained (macroscopic) approximation can be performed by expressing the resulting temporal dynamics as a system-size expansion. The leading order term gives the macroscopic behaviour, while the subsequent orders capture the stochastic nature of the system (for details, see [33]). The resulting macroscopic dynamics follow

$$\frac{\text{d}N^{*}}{\text{d}t}\;=\;N^{*}\left(\alpha g_{b}\left(\frac{N^{*}}{K+f_{1}\left(E\right)}\right)-\delta_{N}g_{d}\left(\frac{N^{*}}{K+f_{1}\left(E\right)}\right)\right)$$ (13)

$$\frac{\text{d}P^{*}}{\text{d}t}\;=\;N^{*}f_{2}\left(E\right)-\delta_{P}P^{*}$$ (14)

$$\frac{\text{d}V^{*}}{\text{d}t}\;=\;V^{*}\left(f_{3b}\left(P^{*}\right)-f_{3d}\left(P^{*}\right)\right).$$ (15)

Systems (13)–(15) describes the evolution in time of the three magnitudes defining the state of the tumour (tumour cells, proteins and vessel cells) in such a way that any tumour (finite *N*, *P*, *V* ≥ 0) will evolve to a steady state (see Theorem A in S1 Text). Thus, to determine and classify the tumour growth dynamics is mandatory to find the steady states of the system ($\dot{N}=\dot{V}=\dot{P}=0$). Different steady states can be associated with different tumour morphologies with differentiated biological consequences.

#### Macroscopic tumour morphologies

The steady states of Eqs (13)–(15) will be labelled alphabetically: *A*, *B* and *C*_*i*_. The trivial steady state

$$A=\left(N_{A}^{*},P_{A}^{*},V_{A}^{*}\right)=\left(0,0,0\right)$$

always exists, and corresponds to the case in which the tumour disappears completely. This state is always unstable (see Lemma D in S1 Text). In the rest of steady states (N* ≠ 0), i.e. the final state of the tumour will always have a finite number of tumour cells so the relative measure *E* = *V*/*N* is mathematically well defined. To study the existence and stability of these solutions comes in handy to rewrite Eq (15) in terms of *E* without any loss of generality

$$\begin{array}{c} \frac{dE}{dt}=\left(f_{3b}\left(P^{*}\right)-f_{3d}\left(P^{*}\right)-\alpha g_{b}\left(\frac{N^{*}}{K+f_{1}\left(E\right)}\right)+\delta_{N}g_{d}\left(\frac{N^{*}}{K+f_{1}\left(E\right)}\right)\right)E. \end{array}$$ (16)

In order to have a finite tumour (N* ≠ 0), a balance of production and death of tumour cells is required (see Eq (13))

$$\begin{array}{c} \alpha g_{b}\left(\gamma \right)=\delta_{N}g_{d}\left(\gamma \right)\;\;\text{with}\;\;\gamma =\frac{N^{*}}{K+f_{1}\left(E\right)} \end{array}$$ (17)

where the solution *γ* is defined uniquely because *g*_*b*_ is a decreasing positive function, *g*_*d*_ is increasing non-negative, *g*_*d*_(0) = 0, and *g*_*b*_(∞) = 0. In particular, if *n*_1_ = *n*_2_, then *γ* is given by the following analytical expression

$$\gamma =\sqrt[{n_{1}}]{\frac{\alpha }{\delta_{N}}}.$$

Similarly, in order to reach the steady state condition *dE*/*dt* = 0 in Eq (16) requires *E* = 0 or a balance in the vessel production and degradation through *f*_3*b*_(P*) = *f*_3*d*_(P*). If *E* = 0, from Eq (17) we obtain N* = *Kγ*, since *f*_1_(0) = 0. Using Eq (14) we get P* = *a*_2_ N*/*δ*_*P*_, obtaining the steady state

$$B=\left(N_{B}^{*},P_{B}^{*},V_{B}^{*}\right)=\left(K\gamma ,a_{2}K\gamma /\delta_{P},0\right).$$

This solution corresponds with the case in which the vessel network disappear from the tumour, while the number of tumour cells reach a constant amount, *i.e*. we have an avascular tumour. The steady state *B* is locally asymptotically stable if the condition *f*_3*b*_(*a*_2_ *Kγ*/*δ*_*P*_) < *f*_3*d*_(*a*_2_ *Kγ*/*δ*_*P*_) holds; for details see Proposition E in S1 Text.

Finally, it only remains to consider the steady state corresponding with *E* ≠ 0, and the vessel balance *f*_3*d*_(P*) = *f*_3*b*_(P*). This last equation has exactly one positive solution denoted by ${\tilde{P}}^{*}$ (see of Fig 1D). Moreover, in the particular case *a*_3_ = *b*_3_ we have ${\tilde{P}}^{*}=m_{3}$. On the other hand, solution to Eq (17) gives N* = *γ*(*K* + *f*_1_(*E*)). Introducing this expression of N* into Eq (14) we obtain

$$\begin{array}{c} f_{2}\left(E\right)\gamma \left(K+f_{1}\left(E\right)\right)=\delta_{P}{\tilde{P}}^{*}. \end{array}$$ (18)

This equation can have multiple solutions, determining the steady states *C*_*i*_. The existence of these steady states correspond with the positive zeros of the auxiliary function

$$\begin{array}{c} h\left(E\right)=f_{2}\left(E\right)\left(K+f_{1}\left(E\right)\right)-\frac{\delta_{P}}{\gamma }{\tilde{P}}^{*}. \end{array}$$ (19)

**Fig 2:**
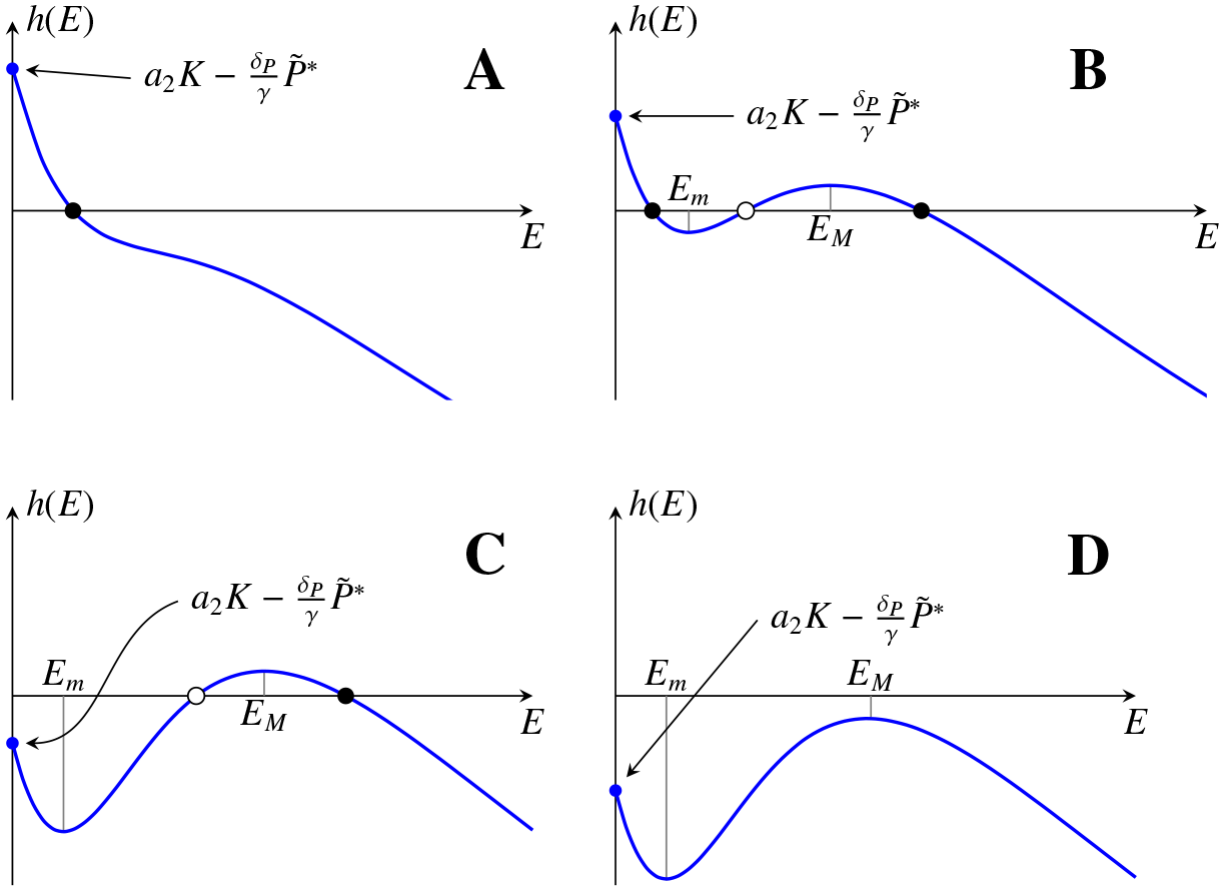
Possible stable morphologies of a vascular tumour. Different possible shapes of the function *h*, which zeros determine the coordinates of positive steady states of systems (13), (14) and (16). The coordinates *E*_*C*_*i*__ of the steady states are indicated with black circles (stable states) or white circles (unstable states). *E*_*M*_ and *E*_*m*_ indicate the values at which *h* reaches its local maximum and minimum.

For the functions *f*_1_ and *f*_2_ given by Eqs (3) and (7), respectively, there can be at most three solutions to Eq (18) (see Fig 2 and Proposition B in S1 Text). The stability of a steady state $C_{i}=\left(N_{C_{i}}^{*},{\tilde{P}}^{*},V_{C_{i}}^{*}\right)$ depends on the behaviour of the function *h* given by Eq (19) at the point $E_{C_{i}}=V_{C_{i}}^{*}/N_{C_{i}}^{*}$. Namely, if the function *h* is increasing in some neighbourhood of *E*_*C*_*i*__, then *C*_*i*_ is unstable (white circles in Fig 2). Otherwise, it is locally asymptotically stable (black circles in Fig 2) (See Proposition G and Remark H in S1 Text for details).

**Fig 3:**
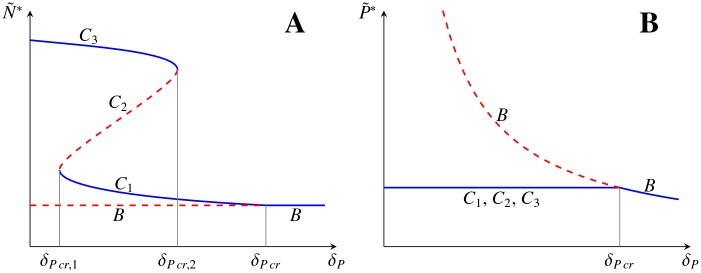
Bifurcation diagram showing coexistence of different tumour morphologies depending on protein degradation rate. Scheme showing a possible bifurcation diagram for the systems (13)–(15) for the tumour cell density N* (A) and the protein density P* (B) as a function of *δ*_*P*_. In the interval *δ*_*Pcr*,1_ < *δ*_*P*_ < *δ*_*Pcr*,2_ (for more details see Proposition B in S1 Text) a coexistence of the stable steady states is observed. Stability is indicated with solid lines (stable solutions) and dashed lines (unstable solutions).

The number of stable states depends on the sign of *h*(0) and the local maximum and minimum values of *h*(*E*), in a generic case, having four different outcomes gathered in Fig 2: No steady state (D); one stable steady state (A); two steady states, one stable and one unstable (C); three steady states, two stable and one unstable (B). In all of them the stability alternates with increasing *E*, being the largest *E*_*C*_*i*__ always stable. Changing the parameters of the model one can change the shape of *h*(*E*) or shift *h*(*E*) vertically, thus changing the number and positions of steady states *C*_*i*_. This is illustrated in Fig 3 where it is indicated the existence and stability of the steady states *C* and *B* in dependence on the parameter *δ*_*P*_.

In Table 2 the existence and stability results of the steady states of the systems (13)–(15) are summarised

**Table 2:** Steady states of the macroscopic systems (13)–(15).

Steady state	Stability
*A* = (0, 0, 0)	unstable
*B* = (*Kγ*, *a*_2_ N*/*δ*_*P*_, 0)	stable for *f*_3*b*_(*a*_2_ *Kγ*/*δ*_*P*_) < *f*_3*d*_(*a*_2_ *Kγ*/*δ*_*P*_)
	unstable for *f*_3*b*_(*a*_2_ *Kγ*/*δ*_*P*_) > *f*_3*d*_(*a*_2_ *Kγ*/*δ*_*P*_)
$\begin{array}{l} C_{i}=\left(N_{C_{i}},{\tilde{P}}^{*},{\tilde{E}}_{C_{i}}N_{C_{i}}\right)\\ \;\;f_{3b}\left({\tilde{P}}^{*}\right)=f_{3d}\left({\tilde{P}}^{*}\right),\\ \;\;h\left(E_{C_{i}}\right)\;=\;0\\ \;\;N_{C_{i}}=\gamma \left(K+f_{1}\left(E_{C_{i}}\right)\right) \end{array}$	stable for *h*′(*E*_*C*_*i*__) < 0
	unstable for *h*′(*E*_*C*_*i*__) > 0

### Stochastic analysis

Dynamical systems (13)–(15) correspond to the macroscopic limit in which the population number is big enough to neglect the intrinsic fluctuations of the system. Nevertheless, there is always a finite population number in a real tumour and the study of stochastic effects is necessary to understand the biological scenario. Fluctuations appear naturally in the definition of the microscopic system (Eqs 1–10).

#### Stochastic description of the model

The exact stochastic dynamics describe a multivariate birth-death process [26] that can be tackled analytically by means of the corresponding Master Equation [33, 34]. Unfortunately, the analytical solution of the Master Equation exists only for a few simple situations, *e.g*. when birth and death are constants and the corresponding Master Equation is linear. This is not true for the current case and numerical simulations of the birth-death process are required. This can be achieved by means of the Gillespie algorithm [35], which generates exact stochastic realisations of the microscopic reactions.

Even though the system can be simulated exactly using Gillespie algorithm, when the number of reactions is high, the simulations can be computationally heavy. Additionally, such simulations do not provide analytical insight into the magnitude of the fluctuations. For this reason it is often useful to approximate the stochastic process, for instance, through state-space truncating procedures [36] or by approximating the fluctuations as a superimposed random diffusion [32, 37]. This latter case includes the Chemical Langevin Equation (CLE), which introduces the fluctuations as a multiplicative noise to the macroscopic evolution of each one of the species in Eqs (13)–(15). The intensity of the noise is determined by each reaction channel rate and its stoichiometry [32–34]. This leads to the following system of stochastic differential equations

$$\begin{array}{c} \begin{array}{cc} \frac{dN^{*}}{dt} & =\left(\alpha g_{b}-\delta_{N}g_{b}\right)N^{*}+\Omega^{-1/2}\sqrt{\left(\alpha g_{b}+\delta_{N}g_{d}\right)N^{*}}\xi_{N}\left(t\right)\\ \frac{dP^{*}}{dt} & =f_{2}N^{*}-\delta_{P}P^{*}+\Omega^{-1/2}\sqrt{f_{2}N^{*}+\delta_{P}P^{*}}\xi_{P}\left(t\right)\\ \frac{dV^{*}}{dt} & =\left(f_{3b}-f_{3d}\right)V^{*}+\Omega^{-1/2}\sqrt{\left(f_{3b}+f_{3d}\right)V^{*}}\xi_{V}\left(t\right). \end{array} \end{array}$$ (20)

By *ξ*_*i*_(*t*) we denote white Gaussian noises with the following properties

$$\langle \xi_{i}\left(t\right)\rangle \;=\;0$$ (21)

$$\langle \xi_{i}\left(t\right)\xi_{j}\left(t^{\prime }\right)\rangle \;=\;\delta \left(t-t^{\prime }\right)\delta_{ij}$$ (21)

where *δ*(*t*−*t*′) denotes the Dirac delta distribution and *δ*_*ij*_ is Kronecker’s delta. The different noise terms in Eq (20) are uncorrelated between themselves because each reaction only involves changes in one of the species.

The CLE is valid as long as a time scale exists such that all the reactions take place without a relevant change in their reaction probability. This is similar to other expansions of volume *Ω* in which the stochastic effect is reduced to an order Ω^1/2^, see *e.g*. [32, 33]. Note that the deterministic limit (Eq 13) is recovered for Ω → ∞.

In the current analysis we use both, the Gillespie algorithm and the Langevin description. The first returns exact trajectories while the second is used to determine analytical properties or obtain stochastic trajectories when the populations of the species is large enough. Actually, later in the text, the Langevin system will be tested for small populations to assess the agreement of both descriptions obtaining that the CLE approximation is enough in most biological relevant tumour scenarios.

#### Behaviour of the stochastic system

**Fig 4:**
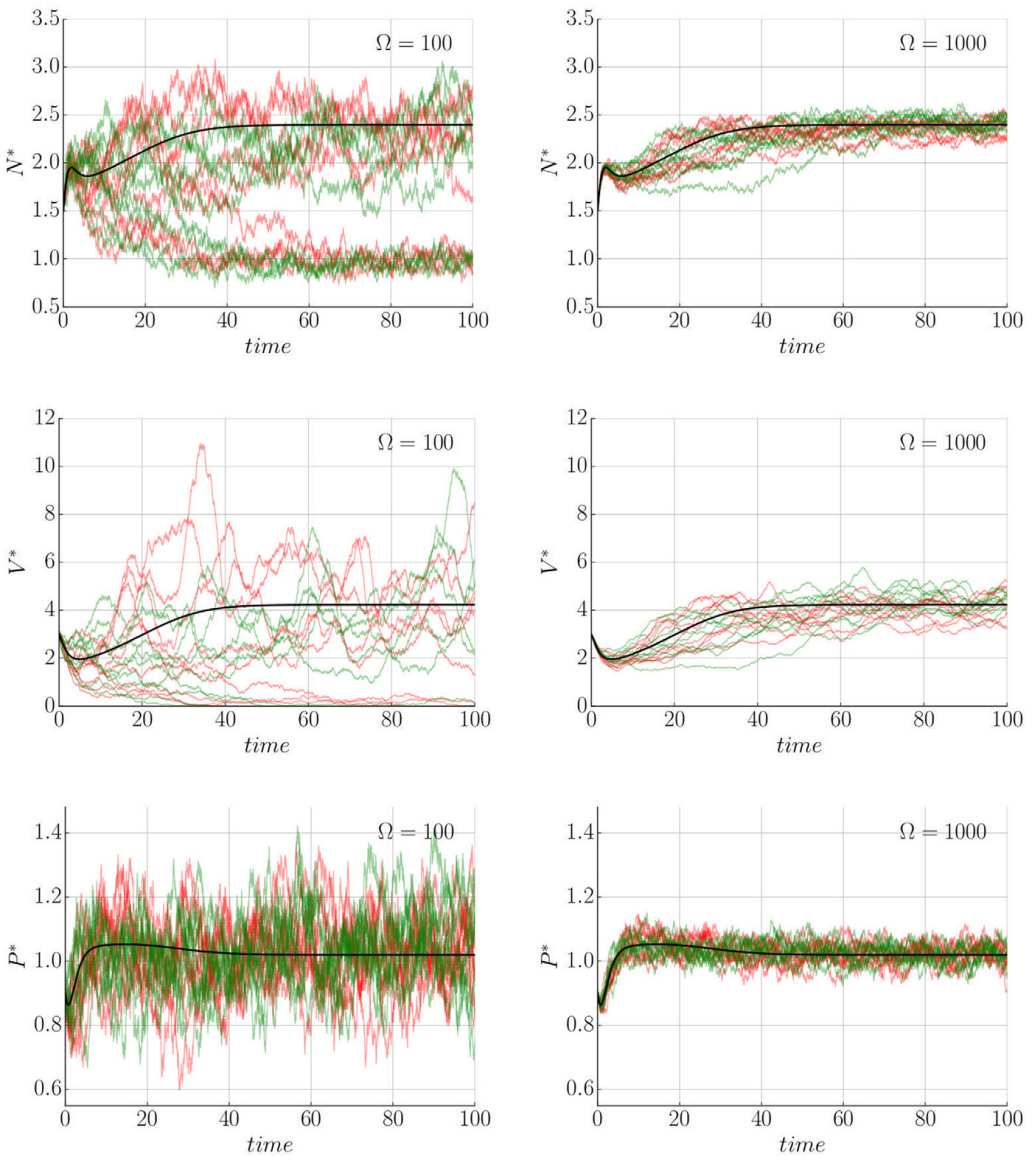
Fluctuations change the macroscopic evolution of the tumour. Deterministic solution (black) compared with 10 different realizations of Gillespie (red) and Langevin (green) for two different values of Ω = 100,1000. Time courses show variability around the macroscopic description and even jumps to different tumour states. Initial condition (1.5, 0.9, 3.0) and parameters are *α* = 1.0, *δ*_*N*_ = 1.1, *δ*_*P*_ = 0.34, *K* = 1.0, *b*_1_ = 2.3, *c*_1_ = 1.5, *a*_2_ = 0.4, *c*_2_ = 1.0, *a*_3_ = 1.0, *m*_3_ = 1.02, *b*_3_ = 1.0, *n* = 2, *n*_1_ = 1, *n*_2_ = 2 and *n*_3_ = *n*_4_ = 2 (see [21]).

**Fig 5:**
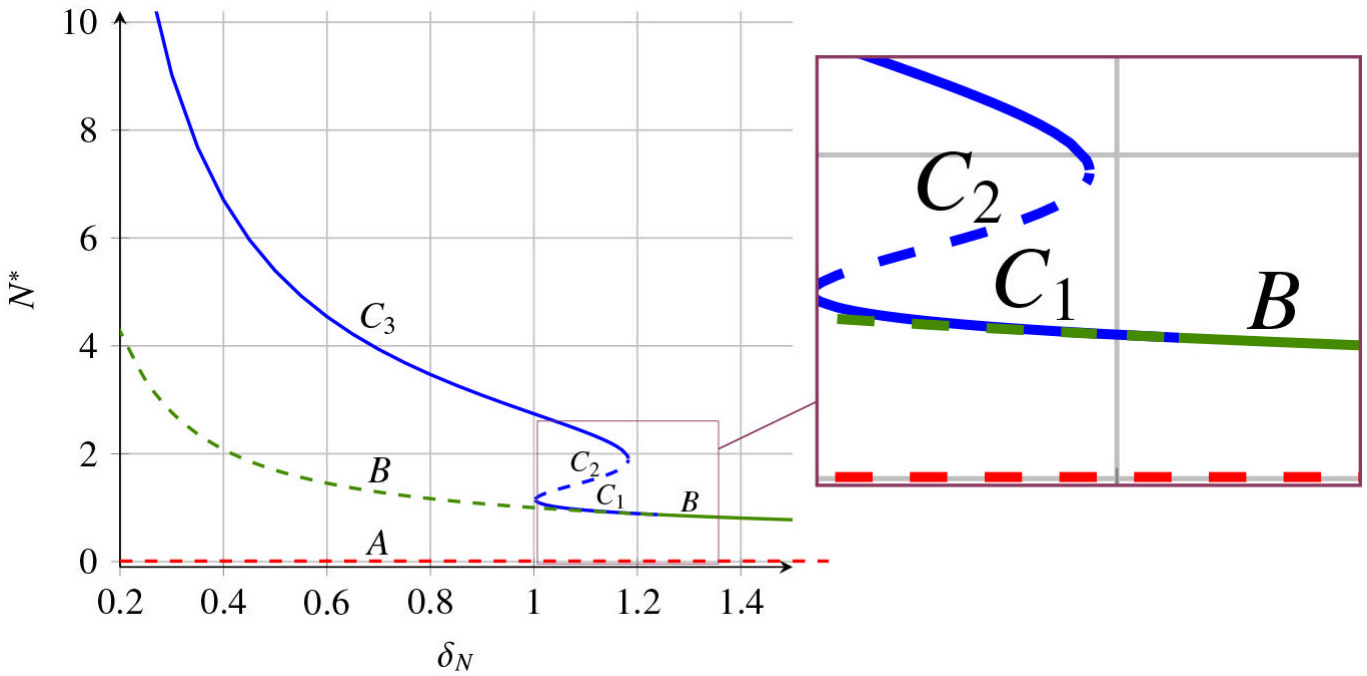
Bifurcation diagram showing coexistence of different tumour morphologies depending on cell death rate. Stability diagram for N* depending on parameter *δ*_*N*_ of systems (13)–(15). Model parameters are as indicated in the caption of Fig 4. Different steady states of the system are drawn with different colours: *A* (red), *B* (green) and *C*_*i*_ (blue). Solid line indicates stability while dashed one instability of the corresponding steady state.

**Fig 6:**
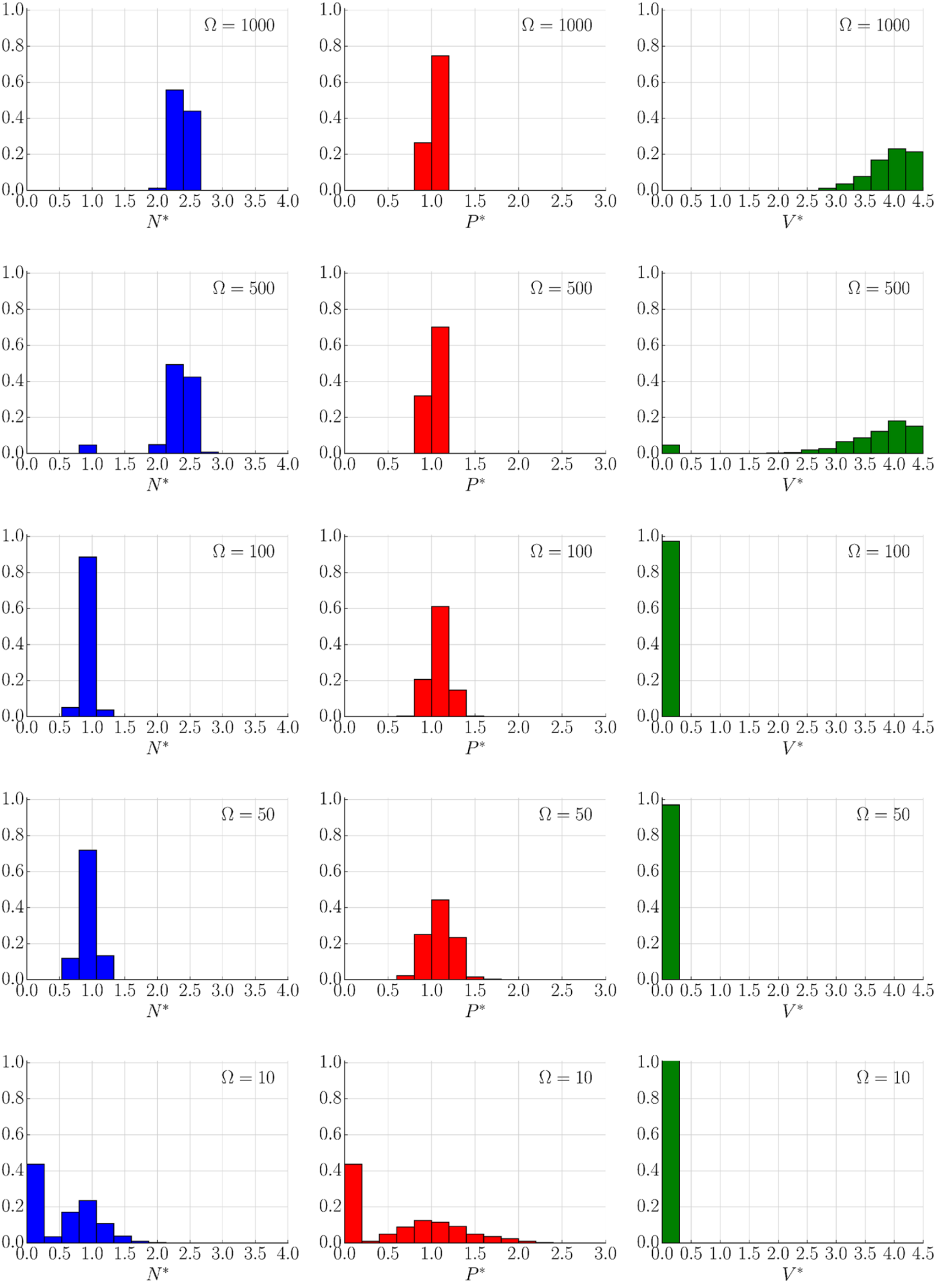
System size determines stochastic populations (Langevin dynamics). Distribution of (N*, P*, V*) at time *t*_total_ = 10000 of 1000 different realizations of Langevin simulation for different values of Ω = 10, 50, 100, 500 and 1000. Initial condition N* = 2.2, P* = 1.0, V* = 3.5 (close to steady stable state *C*_3_). The parameters are those of Fig 4.

**Fig 7:**
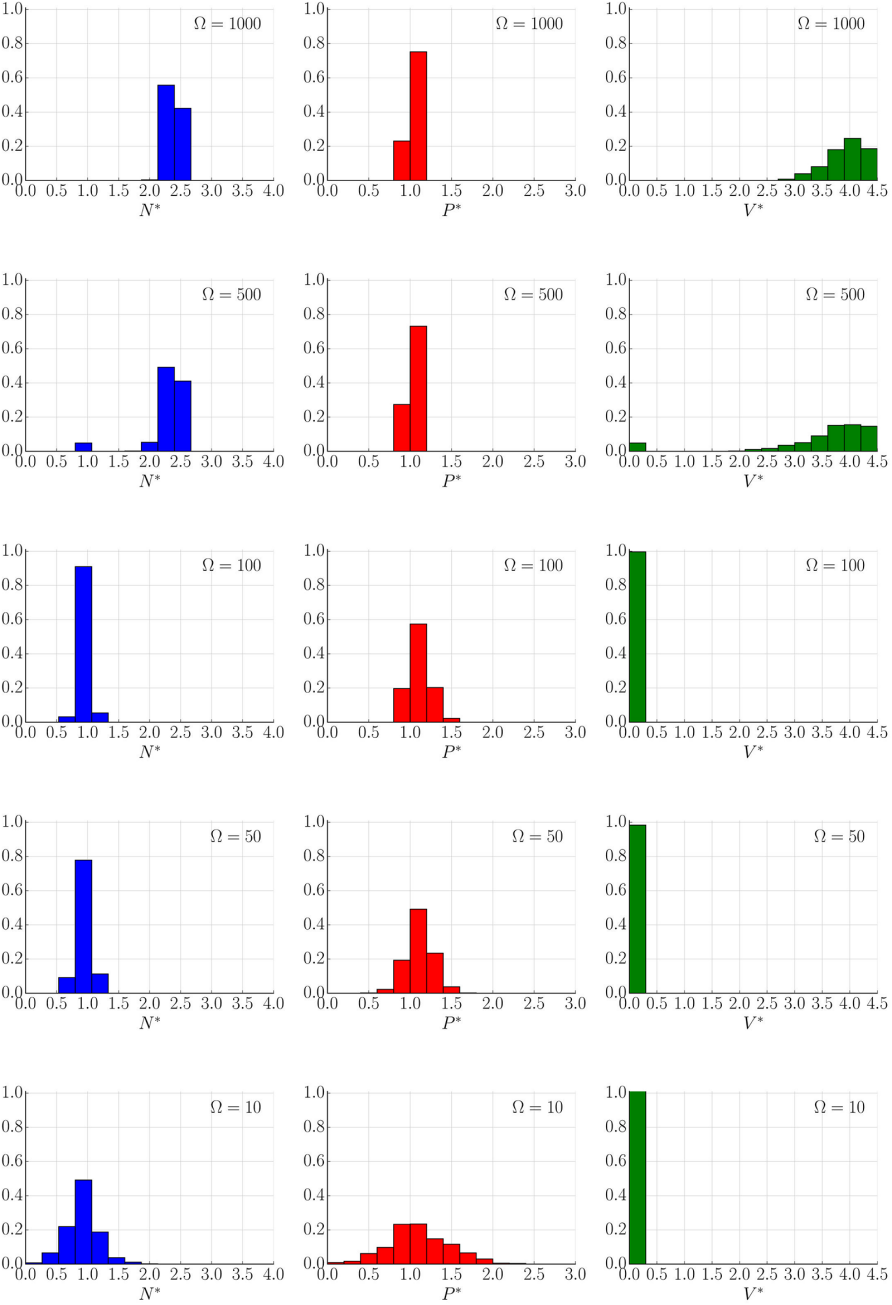
System size determines stochastic populations (Gillespie simulations). Distribution of (N*, P*, V*) at time *t*_total_ = 10000 of 1000 different realizations of Gillespie simulation for different values of Ω = 10, 50, 100, 500 and 1000. Initial condition N* = 2.2, P* = 1.0, V* = 3.5 (close to steady stable state *C*_3_). The parameters are those of Fig 4.

In contrast to the deterministic model, the resulting stochastic trajectories have the ability to explore the whole dynamical landscape. Concretely, one of the effects of the intrinsic noise is the ability to jump between different co-existing stable steady states. This is presented in Fig 4 where the used parameters correspond with previous studies [19, 21] for which the bi-stability behaviour (see Fig 5 and Remark H in S1 Text) is observed, (see S1 Fig). This switching is not available for large Ω (Ω > 1000), where the trajectories follow probability distributions around the corresponding deterministic solution. For smaller volume sizes these transitions become more frequent and the noise plays a more important role determining the final state of the tumour (see Figs 4, 6, 7 and S1 Fig). This effect is also seen as a broadening of the probability distributions around each stable state, providing variability to the tumour characteristic even when it can be described within a single morphology. Additionally, as expected, for very small sizes (Ω ∼ 10) the Langevin approximation can not recapitulate results predicted by the Gillespie algorithm. Concretely, the CLE overestimates the fluctuations giving a higher switching probability. Nevertheless, as will be discussed in the next section, in a biologically relevant scenario Ω ≫ 10. Finally, it is interesting to point out that the transition between states for a growing tumour happens usually at early times when the populations of species is small (Fig 4), even though there is a continuous flux over time that predicts a complete depletion of the macroscopic state for a long enough time (see S1 Fig). A precise quantification of this effect in which jump rate between states changes in time is analysed in the Results section.

Fluctuations also give the system the possibility to visit unstable states such as state *B* for which V* = 0. Nevertheless, once the number of vessel cells reaches zero, the tumour is unable to create more vessels Eq (9). Therefore, the number of vessel cells remains zero during the rest of the evolution of the tumour, describing an avascular state. This can also be predicted from Eq (20), where for *V* = 0 the deterministic and stochastic terms become null. Thus, even though the state *B* can be unstable, it will always capture a stochastic trajectory that reaches *V* = 0 in the absorbent set of states (*N*, *P*, 0).

Likewise, the state *A* = (0, 0, 0) (all the tumour cells disappear) is an absorbent state. Even though state A is linearly unstable, once a fluctuations drives the system in state A cells cannot proliferate and the system is trapped *i.e*. fluctuations can make the tumour disappear. Again, the absorbent state *A*, which is always unstable, has a very different dynamics when the stochastic nature of the tumour is considered.

**Fig 8:**
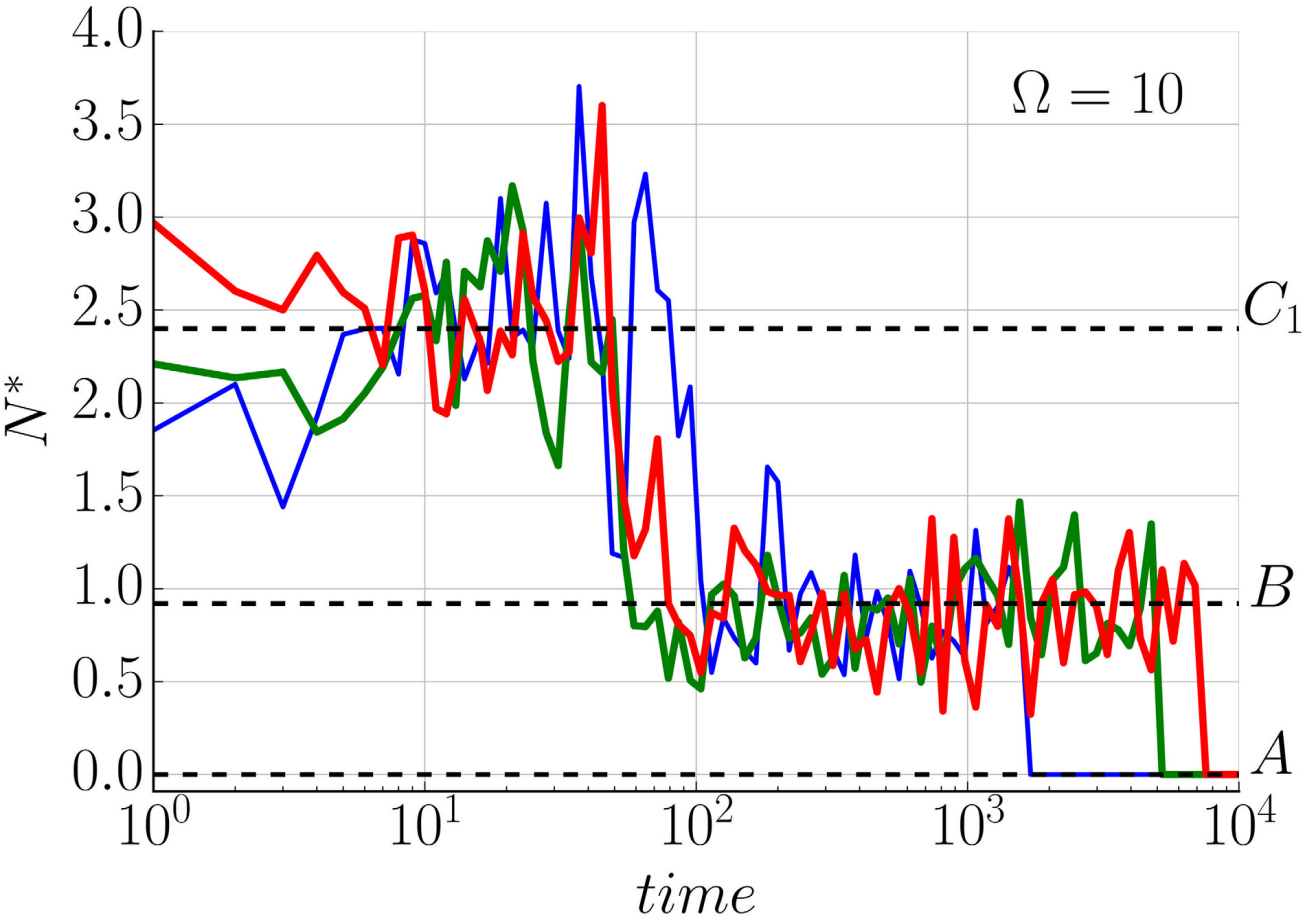
Noise can change the morphology of the tumour over time. Three Langevin trajectories showing a stable tumour becoming avascular and finally disappearing (each trajectory fluctuates around the steady state *C*_3_ then jumps to the vicinity of *B* trapped in the set of states (*N*, *P*, 0) and finally is absorbed by *A*). Initial condition and parameters are those of Fig 3, Ω = 10.

Such behaviour is shown in Fig 8, where a stable tumour becomes avascular and finally dies due to the fluctuations. Since this is an stochastic effect, the rate of absorbency will decrease with increasing Ω and disappear in the deterministic limit (Figs 6 and 7).

### Estimation of the parameters for the Lewis lung carcinoma

The macroscopic models (13)–(15) gives a general description for the evolution of different tumours compatible with the microscopic framework proposed. Nevertheless, the model predictions will vary for different tumour physiologies that come determined by the different parameters that describe the microscopic reactions.

In particular we apply our model to the lung carcinoma (Lewis) mouse tumour for the tumour cell line from [38]. An optimal estimation of model parameters should make use of experiments under identical conditions *e.g*. run by the same lab. Unfortunately, this information is not available, and parameters had to be extracted from different experimental set-ups and sometimes even different mouse lines. Quite often, such experiments only return qualitative data, such as the case of luminescence measurements, and quantitative readouts are only relative.

Under these circumstances, the parameter estimation is driven in two parts. First, by exhausting all the parameters that can be obtained directly from experimental measurements, this first estimation consists of measurements on the VEGF and the tumour cells physiology. This will give a working range of parameters where the final parameter values can be inferred by fitting experimental data on tumour growth to the dynamics predicted by the model.

Since the experimental data provided report the progression of tumour volume (e.g. see [38]), it comes in handy to rewrite the populations in systems (13)–(15) in terms of absolute numbers instead of densities, by rescaling the magnitudes with the parameter Ω without any loss of generality,

$$\begin{array}{c} \begin{array}{cc} \frac{\text{d}N}{\text{d}t}= & N\;\left(\alpha g_{b}\left(\frac{N}{\bar{K}+{\bar{f}}_{1}\left(E\right)}\right)-\delta_{N}g_{d}\left(\frac{N}{\bar{K}+{\bar{f}}_{1}\left(E\right)}\right)\right)\\ \frac{\text{d}P}{\text{d}t}= & N\;f_{2}\left(E\right)-\delta_{P}P\\ \frac{\text{d}V}{\text{d}t}= & V\;\left({\bar{f}}_{3b}\left(P\right)-{\bar{f}}_{3d}\left(P\right)\right) \end{array} \end{array}$$ (23)

where $\bar{K}=K\cdot \Omega$, $\begin{array}{c} {\bar{f}}_{1}\left(E\right)=f_{1}\left(E\right)\cdot \Omega \end{array}$, ${\bar{f}}_{3b}\left(P\right)=f_{3b}\left(P/\Omega \right)$, ${\bar{f}}_{3d}\left(P\right)=f_{3d}\left(P/\Omega \right)$ and ${\bar{m}}_{3}=m_{3}\cdot \Omega$.

#### Estimation of VEGF parameters

The VEGF dynamics comes mainly determined by two parameters, namely, its maximal production rate *a*_2_ and its degradation rate *δ*_*P*_. Nevertheless, VEGF interacts in general through three major isoforms of mouse VEGF (VEGF_120_, VEGF_164_ and VEGF_188_), which have different expression levels depending on the species and tissue type [39] and [40]. For mouse skeletal muscle, the VEGF isoform has been reported to have mRNA expression ratio of (VEGF_164_+VEGF_188_) to VEGF_120_ has been measured to be 92:8, see Results in [40], while for the lung tissue this ratio is 82:18. The VEGF isoform distribution and dynamics has already been studied in a thorough *in silico* model for the mouse skeletal muscle [41], where the secretion rate of VEGF was estimated to be equal to 5875.2 molecules per cell per day; see Results in [41]. Additionally, the lower and upper bounds of the secretion rate of VEGF_164_+VEGF_188_ has been found to be 864 and 17000 molecules per cell per day, respectively. Thus, using the findings on the skeletal muscle mouse with the ratio for lung tissue we can set bounds for total VEGF production rates to be 939 and 19000 molecules per cells per day. Since the estimations made in [41] where done extrapolating information from skeletal muscle tissue to lung tissue we will assume a looser range that will be refined later in the parameter fitting.

On the other hand the VEGF degradation rate *δ*_*P*_ of the three isoforms can be considered comparable and determined from *in vitro* half-life measurements [42, 43], obtaining a degradation rate of 16.6 day^−1^. Again, this datum correspond to a different tissue, in this case human cells. Thus, we treat that estimation as a reference for the mouse tumour.

#### Estimation of tumour cells growth parameters

The maximal tumour growth rate *i.e*. the tumour cell proliferation rate with nutrient profusion has been reported for the lung carcinoma (Lewis) C57BL/6 [44] showing a doubling time of 1–1.7 days. This gives a range for the maximal growth rate parameter *α* in the range 0.41–0.69 day^−1^.

On the other hand, in the absence of nutrient supply from blood vessels the growth of the tumour is hindered reaching a maximal number of tumour cells that in our model comes described by the parameter product $\gamma \bar{K}$. This can be related to the first stages of cancer disease when cells are supplied only by diffusion and no angiogenesis takes place. The typical diameter of the tumour that can be achieved in this avascular phase of growth is 2–3 mm, [1, 2]. Since the average diameter of the Lewis lung cancer (LLC) cell equal to 7 *μm* [38], the average number of cells at the end of avascular growth can be estimated to be in the range 2.33 × 10^7^–7.87 × 10^7^ (cells).

All the estimated parameters above mentioned, their ranges and their bibliographical sources are summarised in Table 3.

**Table 3:** Estimated parameters for Lewis lung carcinoma.

**FITTED PARAMETERS**				
**Parameter**	**Value/Range**	**Unit**	**Ref.**	**Estimated Value**
*a*_2_	939–∞	proteins·cell^−1^·day^−1^	[40, 41]	4.78 × 10^4^
*a*_3_		day^−1^		2.3
*b*_1_		cells		4.48 × 10^9^
*b*_3_		day^−1^		11.5
*c*_1_		—		3.32 × 10^3^
*c*_2_		—		2.38 × 10^−10^
*α*	0.41–0.69	day^−1^	[44]	0.675
*δ*_*N*_		day^−1^		3.58 × 10^−8^
*δ*_*P*_	16.6(*)	day^−1^	[42, 43]	8.91
${\bar{m}}_{3}$		proteins		221
$\bar{K}\cdot \gamma$	(2.33–7.87) × 10^7^	cells	[1, 2, 38]	7.86 × 10^7^
**FIXED PARAMETERS**				
**Parameter**		**Unit**	**Ref.**	**Value**
*N*(0)		cells	[38]	8.29 × 10^8^
*P*(0)		proteins		10
*V*(0)		cells		1
*n*		—		2
*n*_1_ = *n*_2_		—		2
*n*_3_ = *n*_4_		—		2

(*) reference value in the context of the order of magnitude

Note, that due to a lack of experimental data, none of our estimations take into account the number of vessel cells *V*. For instance, in [38] the final vessel cells density for LLC tumours was determined via counting the number of the microvessels per high-power field (HPF) within hot spot area. Similarly, in [45] for the same type of tumour, the number of microvessels were counted for each section and the mean number of microvessels/HPF were calculated as a marker for microvessel density in tumour tissue. In both cases, such data can not be used to interpret the actual number of cell vessels *V*. Similar problems are found in measures of VEGF such as Fig 5B in [45], where the concentration of plasma level of VEGF (expressed in pg/mL) at single time point was measured. Such blood samples were drawn by a terminal cardiac puncture after opening of the chest wall and collected in heparin anti-coagulated tubes preventing to use those data as a measure of VEGF concentration in the tumour tissue.

#### Fitting of the parameters using tumour volume growth data

With the parameter ranges obtained, a further inference can be carried out by using volume data growth for the Lewis lung cancer (LLC) published in [38]. Again, assuming spherical tumour cells of radius *r* = 3.5 *μ*m the volume measurements of LLC cultivated *in vivo* can be directly converted to number of cells. The resulting profile of number of cells in time can be compared with realisations of the model for different parameter values. The optimal values of the parameters can be found by optimising a distance function between the experimental data and the model prediction. In this case we minimise the mean square error (MSE) between both

$$\begin{array}{c} \text{MSE}=\frac{1}{M}\sum_{i=1}^{M}\varepsilon_{i}^{2}\;\text{with}\;\varepsilon_{i}=\frac{x_{i}^{\text{data}}-x_{i}^{\text{sym}}}{x_{i}^{\text{data}}}, \end{array}$$ (24)

where *M* is the number of experimental measurements, $x_{i}^{data}$ are the measured values and $x_{i}^{sym}$ the values of the solution of ODE for respective time points. Due to the number of parameters, the optimisation has been done using Particle Swarm Optimization (PSO), a stochastic optimisation technique that explores efficiently the parameter space [46, 47]. The PSO was carried out using the Matlab^®^ implementation of the algorithm.

**Fig 9:**
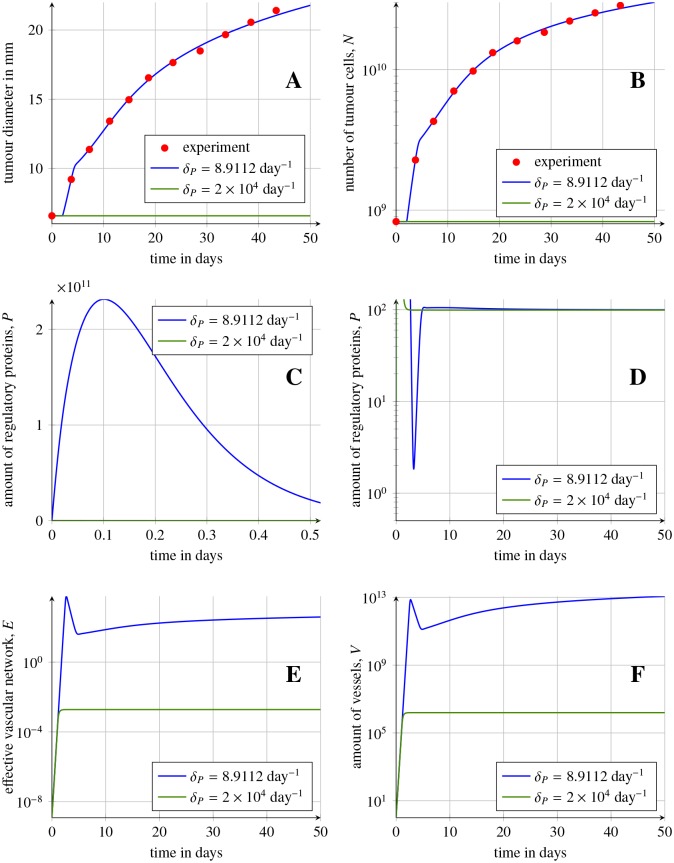
Lewis lung cancer growth prediction during the first month. (A) and (B), comparison of the experimental tumour Lewis lung cancer data from [38] (red circles) and solutions of model (23) for *δ*_*P*_ = 8.9112 day^−1^ (blue lines) for the best fitted parameters, with MSE error equal to 0.07%. (C), (D), (E) and (F), comparison of solutions P*, *E* and V* of system (23) with *δ*_*P*_ = 8.9112 day^−1^ (blue lines). Solid green lines show the behaviour of the model for a high suppression of VEGF (*δ*_*P*_ = 2 × 10^4^ day^−1^). For *δ*_*P*_ > *δ*_*P*_*cr*,2__ the model has only one stable steady state with a small amount of tumour cells. Rest of parameters are given in Table 3.

The resulting optimal set of parameters shows a good agreement with the experimental data (MSE = 0.07%) predicting LLC volume growth during the first months (Fig 9B). The corresponding values of the estimated data are given in Table 3. In order to give soundness to the parameter estimation, a sensitivity study was performed on the inferred parameters. The results of the sensitivity analysis are discussed below in the Results section, while computational details and specific results are gathered in S2 Text.

## Results

The fitted model predicts a single stable state *C* coexisting with unstable states *A* and *B*. Thus, the model suggests that the natural growth of the tumour results as the evolution towards the state *C*, which describes a large tumour with well developed vasculature. This dynamic shows a very rapid secretion of the VEGF proteins within few first hours, that is followed by the fast increase of the vessels network (number of endothelial cells) within few first days of the experiment, see Fig 9. After the first day the active VEGF returns to lower levels. This active VEGF stabilises during the first week causing the future tumour growth. The fast dynamics of VEGF within the first week can be explained by the injection of a large tumour that need to develop vascular network fast.

The performed sensitivity analyses (see S2 Text) shows that after homogeneous perturbations on the parameter set (±2% over each parameter), the resulting perturbed tumour morphology is more sensitive to certain specific parameters. Parameters *δ*_*P*_ and *b*_3_ dominate on determining the number of tumour cells *N* and effective vessel *E*, whereas *m*_3_ and *a*_3_ are also important for determining the number of regulating proteins *P*. It is interesting to point out that the sensitivity of *P* to different parameters strongly changes during the evolution of the tumour. On the other hand, the analysis shows that *K* and tumour cell death rate *δ*_*N*_ are the parameters with less influence *i.e*. the ones with correlation coefficient in the interval (-0.2, 0.2). Including the initial conditions *P*(0) and *E*(0) in the sensitivity analysis (*N*(0) is given by experimental conditions) did not change the sensitivity results (results not shown), suggesting that the results are robust to small perturbations of the initial conditions.

### Intrinsic fluctuations determine the survival of the tumour during the first week

**Fig 10:**
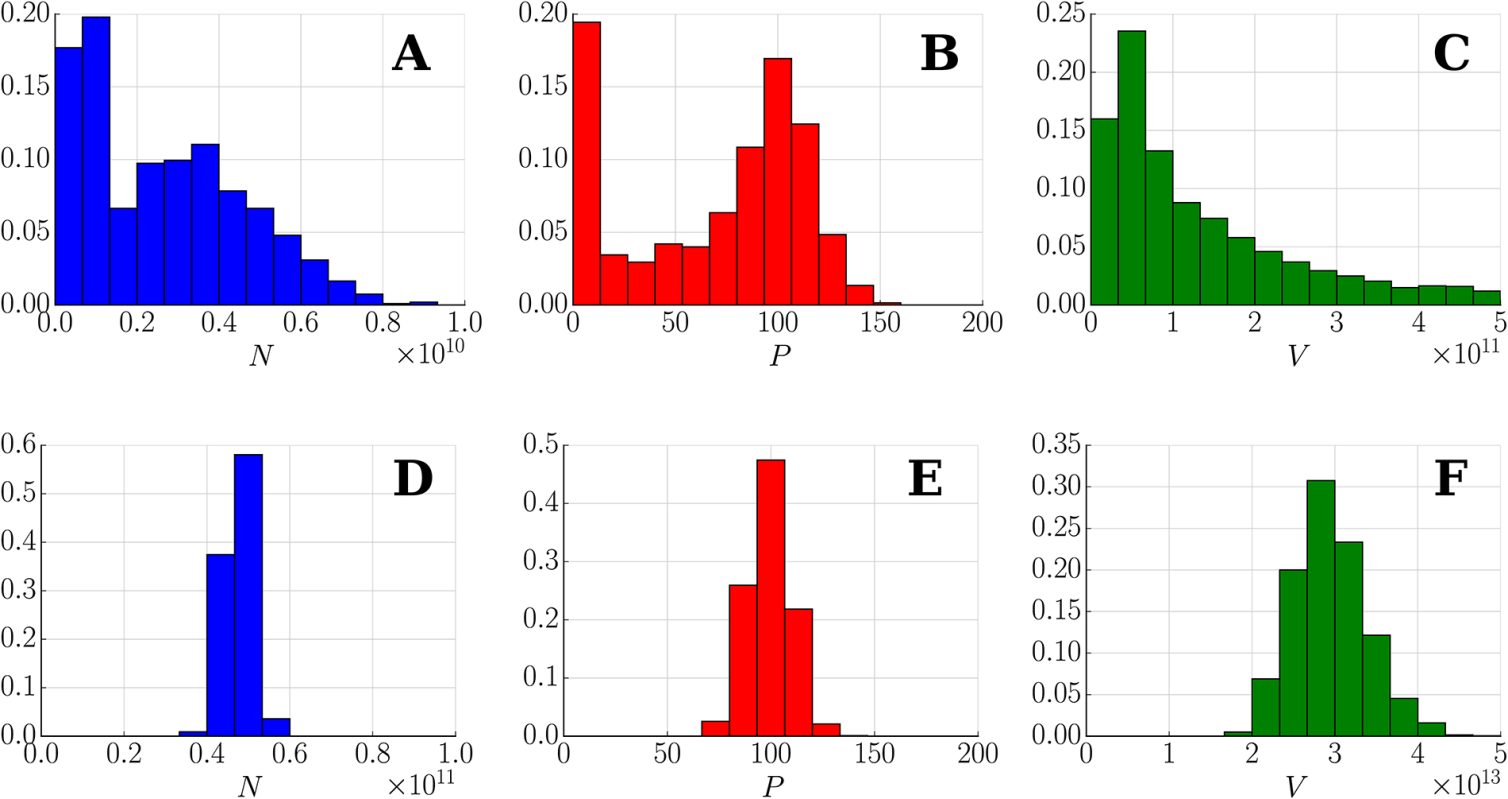
Stochastic population distribution of LLC. Distribution of (*N*, *P*, *V*) for 2000 realizations and parameters of Table 3. (A), (B) and (C): Histogram at time *t* = 5 days. (D), (E) and (F): Histogram at time *t* = 50 days for all the tumours that survived the first week.

The stochastic dynamics of the tumour reveals that the characteristic evolution predicted by the deterministic equations is preserved. Nevertheless, the fluctuation in the population of species can induce on occasions the death of the tumour as described in Materials and Methods where the tumour dynamics is absorbed by the trivial state *A*. For the estimated parameters, the survival of the tumour is determined during the first week when the population of the different species is small (see Fig 10A, 10B and 10C). On the other hand, all the tumours that survive this first week, grow considerably unaffected by the stochastic effects (see Fig 10D, 10E and 10F).

**Fig 11:**
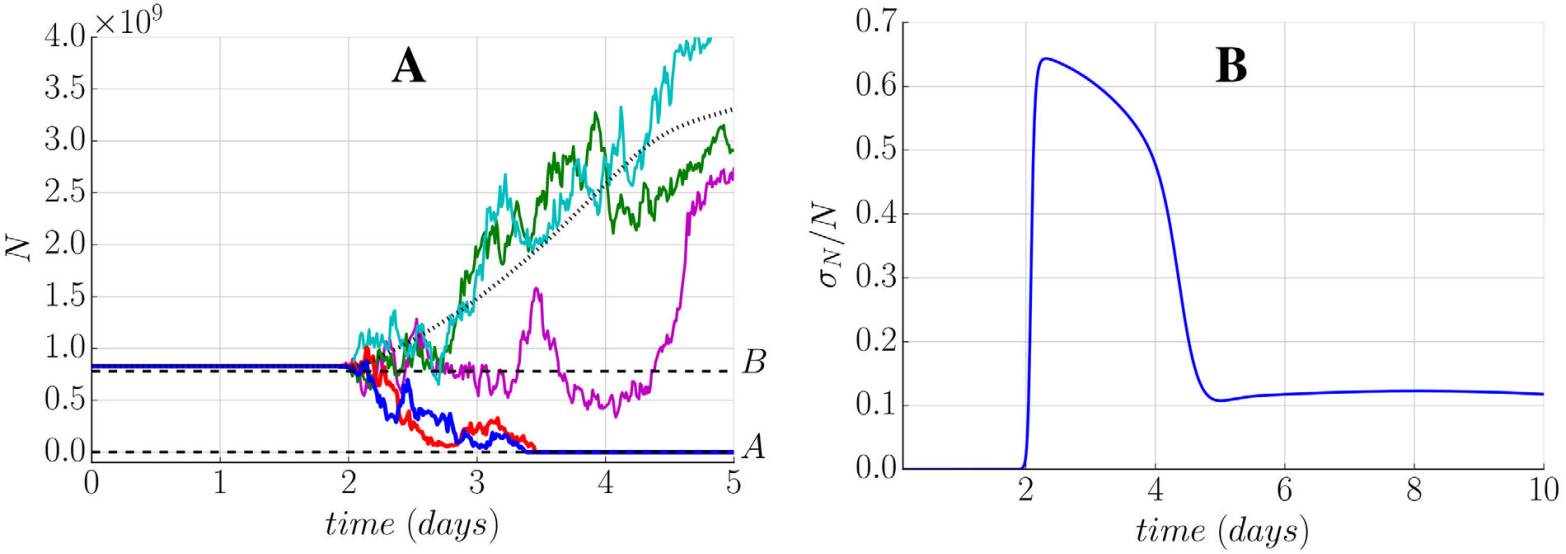
Noise can cause tumour spontaneous extinction during the first week. Stochastic results for the first week growth with parameters of Table 3. (A) Different realizations of the CLE. Two trajectories with a tumour disappearing (begin absorbed by state A), and three trajectories with a successful growth of the tumour, one of them begin transiently trapped around state B. Dotted line shows the deterministic evolution of the tumour. (B) Analytic relative noise intensity given by Eq (20).

A close analysis of the first days of the tumour growth shows that the noise has almost no influence on the dynamics of the model during the first two days (see Fig 11). At the beginning of the second day the noise increases dramatically with two possible outcomes. Either the tumour keeps growing overcoming the noise effects, or a fluctuation drives the tumour to the absorbent state *A*. An analysis of the noise intensity of the CLE Eq (20) can be evaluated analytically along the deterministic trajectory (see Fig 11B). This result points out how the relative intensity of the noise can be neglected during the first two days and grows suddenly until day four were it decays again for the rest of the evolution of the tumour, setting a time window that determines the survival of the tumour. During this time window, around 11% of the growing tumours die spontaneously. This quantity remains practically constant along the rest of the life of the tumour that shows less than a 13% of spontaneous death at day 50 (see S2 Fig). A more detailed analysis (see Remark J in S1 Text) shows that the noise intensity of the tumour cells increases with the effective amount of nutrient *f*_1_(*E*), revealing that the increase in the intensity of the noise between days 2 and 4 is the aftermath of the fast vascularisation predicted during the first days of the tumour growth.

### VEGF degradation rate controls the appearance of dormant tumours

For the parameters inferred, there is only one stable state (*C*). Nevertheless, a further analysis on the dependence of the steady states with biological meaningful changes in the parameters shows that new steady states *C*_*i*_ can arise, for details see S1 Text.

This is the case of VEGF degradation *δ*_*P*_ that can be modified through the administration of a drug (*e.g*. bevacizumab commercial name Avastin), a humanised monoclonal antibody, that binds the vascular endothelial growth factor A (VEGF-A) and thus inhibits VEGF-A binding to receptors on the surface of endothelial cells, effectively increasing *δ*_*P*_.

**Fig 12:**
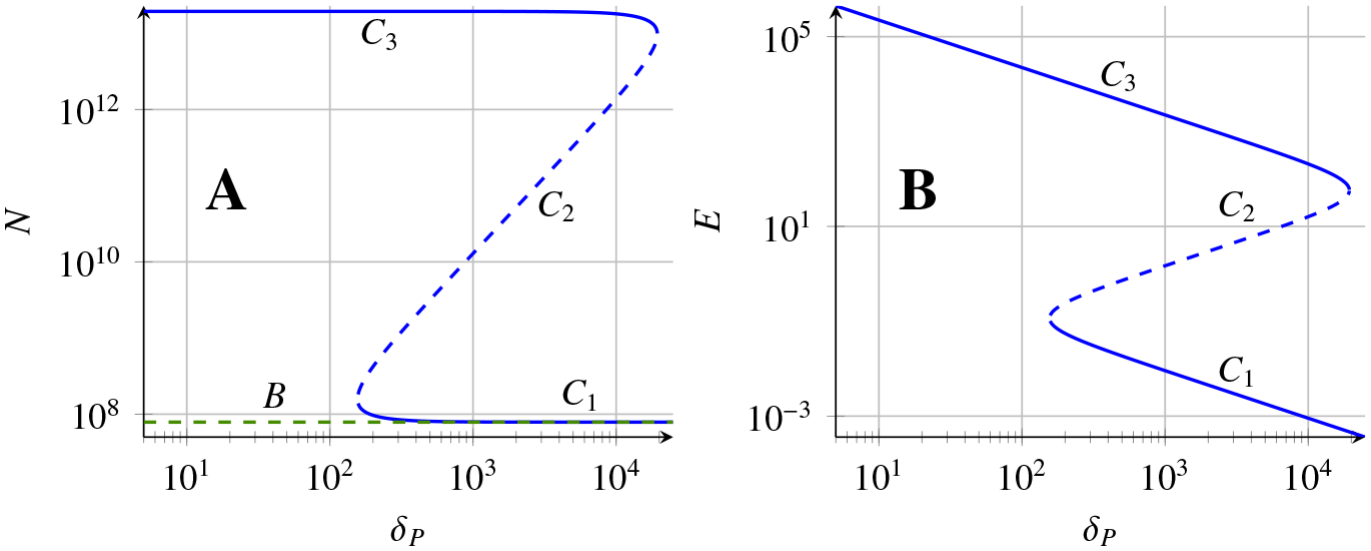
Different tumour morphologies for LLC depending on VEGF degradation rate. Stability diagram for steady states of Eq (23) depending on the parameter *δ*_*P*_, all other parameter values are given in Table 3. The stability of existing steady states in indicated by solid lines, while instability by dashed. Colours indicate the steady states *B* (green) *C*_*i*_ (blue). Additionally, *E* = 0 for state *B*, therefore it is not indicated on the right-hand side panel.

A stability analysis on *δ*_*P*_ reveals that for higher values of *δ*_*P*_ new stable states *C*_*i*_ arise. Concretely, over a threshold *δ*_*Pcr*,1_ ≃ 160 day^−1^ two stable steady *C*_1_, *C*_3_ coexist (see Figs 3 and 12). The new stable tumour morphology *C*_1_ is considerably smaller. Nevertheless, the evolution to this state requires to increase *δ*_*P*_ beyond *δ*_*Pcr*,2_ ≃ 1.2 × 10^4^ day^−1^ where the original state *C*_3_ disappears resulting in a single stable steady state with a comparably smaller amount of tumour cells (see Fig 12 and green lines in Fig 9).

The size of the new tumour morphology *C*_1_ is 8 × 10^7^, corresponding with the number of tumour cells for the maximal tumour size ($\bar{K}\gamma$) that can be achieved without direct nutrient supply from the blood vessels. This is due the fact that for sufficiently large *δ*_*P*_, the number of cells *N* of steady states *C*_1_ and *B* merge (see Fig 12). This result suggests that large enough inhibition of VEGF activity, induces dormant tumours with diameter around 2 mm. Moreover, the effective vessel network (*E*) is of order 10^−4^, that is around nine orders of magnitude lower than the one for the untreated value of *δ*_*P*_ = 8.9 day^−1^, preventing active support of the cancer cells in nutrients and in turn also preventing the fast tumour growth (see Fig 12). Additionally, the stability analysis (which results are summarised in Table 2) shows that the number of proteins in the steady state does not depend on *δ*_*P*_ for the positive steady states *C*_*i*_. For the biologically inferred parameters the number of proteins is low and close to 100 not causing strong stimulation of the endothelial cells, as in the steady state efficient vessel network has been already created and intensive stimulation of the endothelial cells is not needed any more.

## Summary and Discussion

In this paper we have investigated the microscopic foundations of the types of model of tumour angiogenesis proposed in [19, 20] and later studied in [21]. Our model predicts the same qualitative behaviour (regarding the existence and stability of multiple steady states that show hysteresis), being able to reflect the possible instability of the blood vessel formation and structure that is observed in the experiments, see [14, 18], but without the requirement of temporal delays. This was done by describing the tumour dynamics from a set of reactions from which the deterministic macroscopic ODE system was derived. This macroscopic solution predicts different behaviours of the tumour, namely, the tumour disappears (state *A*), the tumour reaches an avascular state (vicinity of state *B*) or the tumour evolves towards a discrete set of tumour morphologies (states *C*_*i*_). This kind of behaviour is not observed in a family of classical Hahnfeldt et al. [3] angiogenesis models without delay where all solutions stabilise at a unique positive steady state. On the other hand, in a modified Hahnfeld et al. model, proposed in [48], there can exist multiple positive steady states, but due to a lack of the hysteresis loop, the solution cannot jump between stable steady states when parameters change.

The microscopic description opens the door to the study of the fluctuations intrinsic to reactive systems. This stochasticity allows the transition between the different tumour steady states, providing new behaviours such as reaching the avascular absorbing set of states (*N*, *P*, 0) or the spontaneous death of the tumour.

In order to explore further the predictions of the model we applied the model to the Lewis lung cancer. The parameters were inferred using the available experimental data in the literature obtaining a good agreement with experimental observations on the tumour growth dynamics in time, during the different phases of tumour formation. A study on the intrinsic noise effects on the tumour dynamics shows that the noise effects are of key importance during the first stage of the tumour formation when fluctuations can destroy the tumour. This stochastic time window of extinction is dictated by the fast vascularisation predicted during the first days of the tumour growth that could be readily tested by a careful tracking of the tumour cell population during this time period. Alternatively, the fast vessel growth could be a consequence of the lack of intervessel competence in Eqs (11) and (12), posing the necessity of such a mechanism for a proper tumour description. In both cases, a deep understanding of this effect and the dependence of this time window with the tumour physiology could provide insight into to tumour prevention and treatment.

We also studied how variations in the degradation of VEGF regulate the tumour progression. The biological *in vivo* degradation rate only predicts one possible tumour state. Nevertheless, the increase of the degradation rate introduces a new stable dormant tumour state that is orders of magnitude smaller. This is biologically relevant since the degradation rate can be easily modified with existing drugs. These two mechanisms of tumour control, spontaneous dead and dormant tumour, are just two cases in which this model helps to determine changes in the tumour morphology that could be addressed experimentally. Nevertheless a more exhaustive analysis may reveal novel strategies in oncology. We hope that this model inspires new experiments and measurements that push forward the knowledge of the field.

Overall, the presented model introduces a simplified reaction scheme being able to exhibit measurable dynamics of tumour growth and vessel formation. Such a reduced model has proved to be complex enough to accommodate experimental data. On the other hand, one can think of a more deeper description of the angiogenesis process described, considering, for instance, vessel cells competition or more complex regulatory functions. However, such approach would require more quantitative data than the one available for the Lewis lung carcinoma. Alternatively, extensions of the model could also address new biological questions such as a spatial description of the angiogenesis process together with the influence of additional vessel growth stimuli factor (e.g. some kinds of interleukins). Moreover, we hope that the general description of the model makes the analysis easily transferable to other types of cancer motivating the use of experimental data in, otherwise, purely theoretical models.

## Supporting Information

S1 Text Mathematical derivation details. Theorems and proofs concerning the existence, uniqueness, boundedness of the solutions; as well as the existence of the positive steady states and conditions for the stability of the steady states for the macroscopic model.
(PDF)

S2 Text Sensitivity analysis of the estimated parameters. Detailed information and results of the sensitivity analysis of the estimated parameters in Table 3.
(PDF)

S1 Fig Tumour cells populations diverges from macroscopic predictions. Histogram showing the probability per trajectory of having N* tumour cells at different times. Results correspond with same parameters used in Fig 4 with 1000 Gillespie realisations. Populations around deterministic state (dark blue) jump over time to a state with less tumour cells (cyan), percentages indicate the probability to find a tumour in each state.
(EPS)

S2 Fig Tumour spontaneous extinction probability is determined during the first week. Histogram showing the probability per trajectory of having N* tumour cells at different times. Results correspond with the same parameters used in Fig 11 with 2000 realisations. A fraction of the growing tumour population (dark blue) dies during the first week *N* = *V* = *P* = 0 (cyan).
(EPS)
