# Supplementary material for: Deterministic and Stochastic Study for a Microscopic Angiogenesis Model: Applications to the Lewis Lung Carcinoma
Source: PLoS One. 2016 May 16;11(5):e0155553. doi: 10.1371/journal.pone.0155553 (PMC4868326; doi:10.1371/journal.pone.0155553)
Supplement: S1 Text — Theorems and proofs concerning the existence, uniqueness, boundedness of the solutions; as well as the existence of the positive steady states and conditions for the stability of the steady states for the macroscopic model. (PDF) [file pone.0155553.s001.pdf]

Deterministic and Stochastic Study for a Microscopic Angiogenesis Model: Applications to the Lewis Lung Carcinoma

Marek Bodnar[1,◑], Pilar Guerrero[2,* ◑], Ruben Perez-Carrasco[2,◑], Monika J. Piotrowska[1,◑]

1 Institute of Applied Mathematics and Mechanics, University of Warsaw, Banacha 2, 02-097 Warsaw, Poland.
2 Department of Mathematics, University College London,Gower Street, London WC1E 6BT, UK.

◑These authors contributed equally to this work and the authors were ordered alphabetically.
*** pguerrero@ucl.ac.uk**

Supporting Information

S1 Text

Derivation of the mathematical results.

Existence, uniqueness and boundedness of the solutions.

Theorem A. *Solutions to system* (13) –(15) *with non-negative initial data exist for all* $t \in \mathbb{R}$*, are unique and the following inequalities*

$$\begin{aligned} 0 &\le N^*(t) \le \min\Big\{N^*(0), \gamma(K + b_1)\Big\}, \\ 0 &\le P^*(t) \le \min\Big\{P^*(0), a_2\gamma(K + b_1)/\delta_P\Big\}, \\ 0 &\le V(t) \le V(0)\exp(b_3 t) \end{aligned} \tag{25}$$

hold for all $t \in \mathbb{R}$*. Moreover, if* $N(0) > 0$*, then there exists* $\varepsilon > 0$ *such that* $N(t) \ge \varepsilon$ *for all* $t \ge 0$*.*

Proof. The local existence and uniqueness of solution follows directly from the right hand-side of (13)–(15), as it is Lipschitz continuous. The non-negativity of N and V follows from writing the appropriate differential equation in an implicit exponential form. In its turn, the non-negativity of P is implied by the non-negativity of N.

From Eq. (13) we deduce that if $N(t) > \gamma(K + f_1(E(t)))$ holds, then $\dot{N}(t) < 0$. Together with the boundedness of the function f_1, this implies the upper boundary of the first inequality (25). A similar argument yields the upper boundary of the second inequation (25). Finally, using the properties of the functions f_{3b} and f_{3d} it can be deduced that $\dot{V} \le b_3 V$ and the upper boundary of the third inequality (25) follows.

Finally we prove that $N(t)$ is bounded away from zero if $N(0) > 0$. Knowing that $N(t) < \gamma K \le \gamma(K + f_1(E(t)))$, we have $\dot{N} > 0$, thus it is enough to take $\varepsilon = \min\{N(0), \gamma K\}$. □

Existence of the positive steady states. The number of positive steady states depends on number of zeros of the function h defined by (19). We have $h(0) = a_2 K - \frac{\delta_P}{\gamma}\tilde{P}^*$ and $h(+\infty) = -\frac{\delta_P}{\gamma}\tilde{P}^* < 0$. Thus, for $a_2 K > \frac{\delta_P}{\gamma}\tilde{P}^*$ there exists at least one positive zero of $h(x)$. Therefore, there exists at least one positive steady state C_1 and in a generic case the number of positive steady states is odd.

Note, that the function h defined by (19) is almost the same as the corresponding function g defined in [21, formula (2.1)] except differences in constants. Below we formulate a proposition, that is an extension of Proposition 2.2 from [21], reformulated in our notation.

Proposition B. *Let f_1 and f_2 be given by* (3) *and* (7)*, respectively.*

1. *For $n = 1$ there can be at most two positive steady states of* (13), (14) *and* (16)*. If $a_2K > \frac{\delta_P}{\gamma}\tilde{P}^*$, then there exist exactly one positive steady state of* (13), (14) *and* (16)*. If $a_2K < \frac{\delta_P}{\gamma}\tilde{P}^*$, then if $c_1 > b_1c_2$ or $h(\bar{x}_0) < 0$, then there exists no positive steady state, while if $h(\bar{x}_0) > 0$, then there exist exactly two positive steady states of* (13), (14) *and* (16)*, where*
$$\bar{x}_0 = \frac{c_1}{b_1+1}\left(\sqrt{b_1\left(\frac{c_2}{c_1}(b_1+1)-1\right)}-1\right). \quad (26)$$

2. *For $n \geq 2$ there can be at most three positive steady states of* (13), (14) *and* (16)*. Moreover,*

   (a) *if*
$$c_1\left(\frac{K}{b_1c_2}\right)^n < \left(1-\frac{1}{n}\right)^n\frac{1}{n-1} \quad (27)$$
   then there exist $0 < \delta_{P\,cr,1} < \frac{a_2\gamma K}{\tilde{P}^*} < \delta_{P\,cr,2} < \frac{a_2\gamma(K+b_1)}{\tilde{P}^*}$ such that for $0 < \delta_P < \delta_{P\,cr,1}$ there exits exactly one positive steady state of (13), (14) *and* (16)*, for $\delta_{P\,cr,1} < \delta_P < \frac{a_2\gamma K}{\tilde{P}^*}$ there exist exactly three positive steady states of* (13), (14) *and* (16)*, for $\frac{a_2\gamma K}{\tilde{P}^*} < \delta_P < \delta_{P\,cr,2}$ there exist exactly two positive steady states of* (13), (14) *and* (16)*, and for $\delta_P > \delta_{P\,cr,2}$ there are no positive steady states of* (13), (14) *and* (16).

   (b) *if reverse inequality to* (27) *holds then for $\delta_P > \frac{a_2\gamma K}{\tilde{P}^*}$ there are no positive steady states of* (13), (14) *and* (16)*, and either there is exactly one positive steady states of* (13), (14) *and* (16) *for all $0 < \delta_P < \frac{a_2\gamma K}{\tilde{P}^*}$ (when the function $h(x)$ is strictly monotonic) or there exist $0 < \delta_{P\,cr,1} < \delta_{P\,cr,2} < \frac{a_2\gamma K}{\tilde{P}^*}$ such that for $0 < \delta_P < \delta_{P\,cr,1}$ and $\delta_{P\,cr,2} < \delta_P < \frac{a_2\gamma K}{\tilde{P}^*}$ there exists exactly one positive steady states of* (13), (14) *and* (16) *and for $\delta_{P\,cr,1} < \delta_P < \delta_{P\,cr,2}$ there exist exactly three positive steady states of* (13), (14) *and* (16).

Proof. The part (i) was proved in [21] as well as the fact that for $n \geq 2$ at most three positive steady states of (13), (14) and (16) can exist.

To prove (ii.a) and (ii.b) note, that the function h given by (19) for $n \geq 2$, can be rewritten as
$$h(x) = -\frac{h_1(x)}{(c_2+x)(c_1+x^n)},$$
where
$$h_1(x) = \tilde{\delta}_P x^{n+1} + c_2\Big(\tilde{\delta}_P - a_2(K+b_1)\Big)x^n + \tilde{\delta}_P c_1 x + c_1c_2(\tilde{\delta}_P - a_2K), \quad \tilde{\delta}_P = \frac{\delta_P}{\gamma}\tilde{P}^*$$
Note also, that the shape of the function h does not change with changing δ_P (or $\tilde{\delta}_P$). Moreover,
$$c_2\Big(\tilde{\delta}_P - a_2(K+b_1)\Big) > 0 \implies c_1c_2(\tilde{\delta}_P - a_2K) > 0 \quad (28)$$
and
$$c_1c_2(\tilde{\delta}_P - a_2K) < 0 \implies c_2\Big(\tilde{\delta}_P - a_2(K+b_1)\Big) < 0. \quad (29)$$
Thus, the number of sign changes of the coefficients of the polynomial h_1 can be:

1. zero, if $\tilde{\delta}_P > a_2(K+b_1)$;
2. two, if $a_2K < \tilde{\delta}_P < a_2(K+b_1)$;

3. three, if $\tilde{\delta}_P < a_2K$.

Using Descart's rule of signs we deduce that in the first case there are no positive roots of h_1, in the second case there can be two or zero positive roots of h_1, and in the third case there can be one positive root or three positive roots of h_1. The same holds for h. As the shape of h does not change with changing $\tilde{\delta}_P$ and the number of roots of h_1 is the same as h, hence, two positive roots of h exist for $a_2K < \tilde{\delta}_P < a_2(K + b_1)$ if and only if in the limiting case, that is for $\tilde{\delta}_P = a_2K$, there exist two strictly positive roots of h_1. For $\tilde{\delta}_P = a_2K$ the function h_1 reads

$$h_1(x)\Big|_{\tilde{\delta}_P=a_2K} = a_2(Kx^n - b_1c_2x^{n-1} + c_1K)x.$$

Two strictly positive roots of $h_1(x)\Big|_{\tilde{\delta}_P=a_2K}$ exist if and only if

$$\min_{x>0}\Big\{Kx^n - b_1c_2x^{n-1} + c_1K\Big\} < 0. \tag{30}$$

Calculating derivative we deduce that the maximum of $Kx^n - b_1c_2x^{n-1} + c_1K$ is reached at $x_{\min} = \left(1 - \frac{1}{n}\right)\frac{b_1c_2}{K}$ and therefore, solving (30) we obtain (27). This, together with implications (28), (29), and Descart's rule of signs proves part (ii) of Proposition B. □

Remark C. *One can easily find that*

$$h'(x) = -\frac{(b_1 + K)x^{2n} - c_1\Big(b_1(n-1) - 2K\Big)x^n - nb_1c_1c_2x^{n-2} + c_1^2K}{(c_1 + x)^2(c_2 + x)^2}$$

and deduce that $h'(0) < 0$ *and it can have zero or two positive roots. In the latter case, the critical value of* $\delta_{P\,cr,j}$, $j = 1, 2$ *can be found calculating zeros of* h', *that is* $0 < x_1 < x_2$ *such that* $h'(x_1) = h'(x_2) = 0$ *and than the critical value of* $\delta_{P\,cr,j}$ *is determined by solutions of equations* $h(x_j) = 0$ *with respect to* δ_P *for each* $j = 1, 2$. *In Fig. 2 we illustrate possible shapes of the function h.*

Although, in a general case it is not possible to derive analytical expressions for $\delta_{P\,cr,1}$ and $\delta_{P\,cr,2}$, having particular model parameters we are able to compute these critical values of δ_P, and determine if we observe the hysteresis loop in the system or not.

Summarising, system (13), (14) and (16) has from one to four non-negative steady state with first coordinate greater then zero. There is exactly one steady state with the last coordinate equal to zero

$$B = (\gamma K, a_2\gamma K/\delta_P, 0)\,,$$

and from zero to three positive steady states

$$C_i = (\tilde{N}_i^*, \tilde{P}^*, \tilde{E}_i)\,, \quad h(\tilde{E}_i) = 0, \;\; \tilde{N}^* = \gamma(K + f_1(\tilde{E})).$$

In order to study stability of the steady state we rescale the model to reduce the number of parameters making the following change of variables

$$\begin{aligned}
&x = \frac{N^*}{K\gamma}, && y = \frac{P^*}{\tilde{P}^*}, && z = E,\\
&s = \delta_P t, && \tilde{f}_2(x) = \frac{K\gamma}{\delta_P\tilde{P}^*}f_2(x), && \tilde{g}_b(x) = \tilde{\alpha}g_b(\gamma x),\\
&\tilde{g}_d(x) = \tilde{\delta}_N g_d(\gamma x), && \tilde{f}_{3b}(x) = \frac{1}{\delta_P}f_{3b}(\tilde{P}^*x), && \tilde{f}_{3d}(x) = \frac{1}{\delta_P}f_{3d}(\tilde{P}^*x),\\
&\tilde{\alpha} = \frac{\alpha}{\delta_P}, && \tilde{\delta}_N = \frac{\delta_N}{\delta_P}, && \tilde{f}_1(x) = \frac{1}{K}f_1(x).
\end{aligned} \tag{31}$$

In the new variables Eq. (13), (14) and (16) read

$$\begin{aligned}
\frac{\mathrm{d}x}{\mathrm{d}s} &= x\left(\tilde{g}_b\Big(\frac{x}{1+\tilde{f}_1(z)}\Big) - \tilde{g}_d\Big(\frac{x}{1+\tilde{f}_1(z)}\Big)\right),\\
\frac{\mathrm{d}y}{\mathrm{d}s} &= x\tilde{f}_2(z) - y,\\
\frac{\mathrm{d}z}{\mathrm{d}s} &= \left(\tilde{f}_{3b}(y) - \tilde{f}_{3d}(y) - \tilde{g}_b\Big(\frac{x}{1+\tilde{f}_1(z)}\Big) + \tilde{g}_d\Big(\frac{x}{1+\tilde{f}_1(z)}\Big)\right)z.
\end{aligned} \tag{32}$$

and the steady states reads (we have $\tilde{g}_d(1) = \tilde{g}_b(1)$ and $\tilde{f}_{3d}(1) = \tilde{f}_{3b}(1)$):

$$B = (1, \tilde{a}_2, 0), \quad \tilde{a}_2 = \frac{a_2 K\gamma}{\delta_P \tilde{P}^*},$$
$$C_i = (1 + f_1(\bar{z}_i), 1, \bar{z}_i),$$

where $\bar{z}_i$ are solution to

$$\tilde{h}(z) = \tilde{f}_2(z)\big(1 + \tilde{f}_1(z)\big) - 1 = 0.$$

Stability of steady states. Note, that for N^* close to 0 we have $\mathrm{d}N^*/\mathrm{d}t > 0$, therefore the following Lemma holds.

Lemma D. *The steady state* $(0, 0, 0)$ *of the system* (13)–(15) *is unstable for arbitrary set of parameters.*

Linearising system (32) around steady state we get stability matrices J. For the steady state B the stability matrix reads

$$J(B) = \begin{bmatrix} d_1 & 0 & -\tilde{f}_1'(0)d_1 \\ \tilde{a}_2 & -1 & \tilde{f}_2'(0) \\ 0 & 0 & \tilde{f}_{3b}(\tilde{a}_2) - \tilde{f}_{3d}(\tilde{a}_2) \end{bmatrix},$$

where $d_1 = \tilde{g}_b'(1) - \tilde{g}_d'(1) < 0$ due to the fact that g_b is decreasing and g_d is increasing. We easily deduce that the steady state B is locally asymptotically stable if $\tilde{f}_{3b}(\tilde{a}_2) < \tilde{f}_{3d}(\tilde{a}_2)$ and is unstable if this inequality is reverse.

Thus we may formulate

Proposition E. *If* $f_{3b}(a_2K\gamma/\delta_P) < f_{3d}(a_2K\gamma/\delta_P)$ *then the steady state* B *of system* (13), (14) *and* (16) *is locally asymptotically stable. On the other hand, if* $f_{3b}(a_2K\gamma/\delta_P) > f_{3d}(a_2K\gamma/\delta_P)$ *then the steady state* B *of system* (13), (14) *and* (16) *is unstable.*

For the steady states C_i we have

$$J(C_i) = \begin{bmatrix} d_1 & 0 & -\tilde{f}_1'(\bar{z}_i)d_1 \\ \tilde{f}_2(\bar{z}_i) & -1 & (1+\tilde{f}_1(\bar{z}_i))\tilde{f}_2'(\bar{z}_i) \\ -d_1\frac{\bar{z}_i}{1+\tilde{f}_1(\bar{z}_i)} & \big(\tilde{f}_{3b}'(1) - \tilde{f}_{3d}'(1)\big)\bar{z}_i & \frac{\bar{z}_i}{1+\tilde{f}_1(\bar{z}_i)}\tilde{f}_1'(\bar{z}_i)d_1 \end{bmatrix},$$

and the characteristic polynomial has the following form

$$W(\lambda) = \lambda^3 + w_2\lambda^2 + w_1\lambda + w_0 = 0,$$

where

$$w_2 = 1 - d_1\left(1 + \frac{\bar{z}_i}{1+\tilde{f}_1(\bar{z}_i)}\tilde{f}_1'(\bar{z}_i)\right), \tag{33}$$

$$w_1 = -d_1\left(1 + \frac{\bar{z}_i}{1+\tilde{f}_1(\bar{z}_i)}\tilde{f}_1'(\bar{z}_i)\right) - d_3\bar{z}_i\big(1+\tilde{f}_1(\bar{z}_i)\big)\tilde{f}_2'(\bar{z}_i), \tag{34}$$

$$w_0 = d_1 d_3 \bar{z}_i\big(\tilde{f}_1'(\bar{z}_i)\tilde{f}_2(\bar{z}_i) + (1+\tilde{f}_1(\bar{z}_i))\tilde{f}_2'(\bar{z}_i)\big), \tag{35}$$

with

$$d_3 = f'_{3b}(1) - f'_{3d}(1) > 0,$$

as f_{3b} is increasing and f_{3d} is decreasing.

Lemma F. *Let $\tilde{h}(\bar{z}) = 0$. Then the sign of $\tilde{f}'_1(\bar{z})f_2(\bar{z}) + (1 + \tilde{f}_1(\bar{z}))\tilde{f}'_2(\bar{z})$ is the same as the sign of $\tilde{h}'(\bar{z})$.*

Proof. The proof follows ideas from [19].

$$\tilde{f}'_1(\bar{z})\tilde{f}_2(\bar{z}) + (1 + \tilde{f}_1(\bar{z}))\tilde{f}'_2(\bar{z}) > 0 \Longleftrightarrow \Big(\ln(1 + \tilde{f}_1(\bar{z}))\Big)' > -\Big(\ln \tilde{f}_2(\bar{z})\Big)'.$$

Thus the above inequalities are equivalent to $(\ln \tilde{h}(\bar{z}))' > 0$. Since logarithm is an increasing function, that means that $\tilde{h}$ increases in the neighbourhood of $\bar{z}$ and therefore $h'(\bar{z}) > 0$. □

Proposition G. *If $h'(\bar{z}_i) < 0$ then the steady state C_i is locally asymptotically stable, and if $h'(\bar{z}_i) > 0$ it is unstable.*

Proof. The Ruth–Hurwitz criterion implies that steady state C_i is locally asymptotically stable if and only if $w_0 > 0$, $w_2 > 0$, and $w_1 w_2 > w_0$. The inequality $d_1 < 0$ implies $w_2 > 0$. The assumptions of Proposition, together with Lemma F imply $w_0 > 0$.

It remains to prove that $w_1 w_2 - w_0 > 0$. In fact, algebraic calculation leads to

$$\begin{aligned} w_1 w_2 - w_0 =& (1 - d_1\eta)\Big(d_3 \bar{x}_i \bar{z}_i |\tilde{f}'_2(\bar{z}_i)| - d_1(1 + \eta)\Big) + \\ & - d_1 d_3 \bar{z}_i \tilde{f}'_1(\bar{z}_i)\tilde{f}_2(\bar{z}_i) + (1 + \eta) d_3 \bar{x}_i \bar{z}_i |\tilde{f}'_2(\bar{z}_i)|, \end{aligned}$$

with $\bar{x}_i = 1 + \tilde{f}_1(\bar{z}_i)$ and $\eta = \bar{z}_i \tilde{f}'_1(\bar{z}_i)/\bar{x}_i$. It is ease to see that the above expression is positive under the assumption of Proposition. □

Remark H. *Note, the stability of the steady states C_i follows the same pattern as in [19] and [21]. Thus, if there exists three positive steady states, then those with the lowest and the highest number of tumour cells are stable while the third one is unstable, see Fig. 3. This means that it is possible to observe the hysteresis effect in the model when one changes one of the model parameters and keeps other parameters unchanged.*

Remark I. *The condition for the stability of the steady state B for $n_3 = n_4$ can be rewritten as*

$$\delta_P > \frac{a_2\gamma K}{\tilde{P}^*} = \frac{a_2\gamma K}{m_3}\sqrt[n_3]{\frac{b_3}{a_3}}$$

because for $x = m_3 \sqrt[n_3]{a_3/b_3}$ we have the intersection point of curves given by $f_{3b}(x)$ and $f_{3d}(x)$.

Dependence of the noise intensity with the effective amount of nutrient.

Remark J. *The noise intensity for the evolution of tumour cells σ_N/N predicted by the CLE 20 is,*

$$\frac{\sigma_N}{N} = \alpha g_b(x) + \beta g_d(x) = \frac{\alpha + \beta x^2}{1 + x^2} \tag{36}$$

where $x = N^*/(K + f_1(E))$, and the condition from Table 3, $n_1 = n_2 = 2$ has been used. The resulting noise intensity (36) is a function that decreases monotonically with x reaching a maximum at $x = 0$. Thus, for a constant value of tumour cells N, the noise intensity will increase with the effective amount of nutrient $f_1(E)$. This is of special relevance during the initial days, where a fast vascularisation entails a dramatic increase of the fluctuations in the number of tumour cells.
