# Supplementary material for: Deterministic and Stochastic Study for a Microscopic Angiogenesis Model: Applications to the Lewis Lung Carcinoma
Source: PLoS One. 2016 May 16;11(5):e0155553. doi: 10.1371/journal.pone.0155553 (PMC4868326; doi:10.1371/journal.pone.0155553)
Supplement: S2 Text — Detailed information and results of the sensitivity analysis of the estimated parameters in Table 3. (PDF) [file pone.0155553.s002.pdf]

Deterministic and Stochastic Study for a Microscopic Angiogenesis Model: Applications to the Lewis Lung Carcinoma

Marek Bodnar[1,◑], Pilar Guerrero[2,*,◑], Ruben Perez-Carrasco[2,◑], Monika J. Piotrowska[1,◑]

1 Institute of Applied Mathematics and Mechanics, University of Warsaw, Banacha 2, 02-097 Warsaw, Poland.
2 Department of Mathematics, University College London,Gower Street, London WC1E 6BT, UK.

◑These authors contributed equally to this work and the authors were ordered alphabetically.
*** pguerrero@ucl.ac.uk**

Supporting Information

S2 Text

Sensitivity analysis of the estimated parameters

Initial parameters ranges obtained from experimental data in the bibliography are not known with high accuracy, some of them having an initial uncertainty of one or more orders of magnitude. For this reason it is interesting to study the effect of perturbations in the estimated parameters on the predicted behaviour of the model. An evaluation of the partial derivative of the solution with respect to each parameter, only provides information about the effect of a small variation in each parameter separately about its nominal value and it does not indicate the effect of perturbations of the whole parameter ensemble. This problem can be overcome by performing a global sensitivity analysis in order to find correlations between the model behaviour and model parameters. This also allows us to find which parameters have the strongest influence on the solution of the model. To this end, 4000 independent sets of parameters (11 in total, keeping all Hill coefficients fixed $n_i = 2$) were generated using the standard sampling technique Latin Hypercube Sampling, which based on a probability density function (in our case for all parameters we take uniform distributions with $\pm 2\%$ uncertainty interval) allows to divide the whole domain in equiprobable serial intervals. A single value is selected randomly from every interval, allowing us to use an input value from each sampling interval only once in the analysis, while sampling the parameter space evenly. The sensitivity analysis was performed on the deterministic model using the freely downloadable SaSAT (Sampling and Sensitivity Analysis Tools) software, see [Hoare *et.al.* Theo. Bio. Med. Mod. (2008)], which also includes more details on the sampling algorithm.

We studied the influence of the model parameters on the tumour morphology at three time points of the tumour evolution. Examining the resulting partial rank correlation coefficients it is observed that the most correlated parameters with the the predicted tumour morphology are b_3 and δ_P, see Fig. S2.1 for details. The corresponding scatter plots comparing the number of cells/proteins and effective vessel network against the selected parameters, for the last measurement time, also show this strong positive/negative correlation (see Fig. S2.2).

An alternative measure for the sensitivity is the factor of prioritisation, comparing the parameters by the corresponding effect on the output variances, see Fig. S2.3. The calculated sensitivity index can be interpreted as the proportion of the total variance of the result attributable to each considered input parameter in such a way that, the higher the index value is, the more sensible the considered model is to that specific parameter. The sensitivity index for

which the adjusted R^2 is close to one indicates a good accuracy of the results. As it can be seen, these results agree with the partial rank correlation coefficients showing similar predictions.

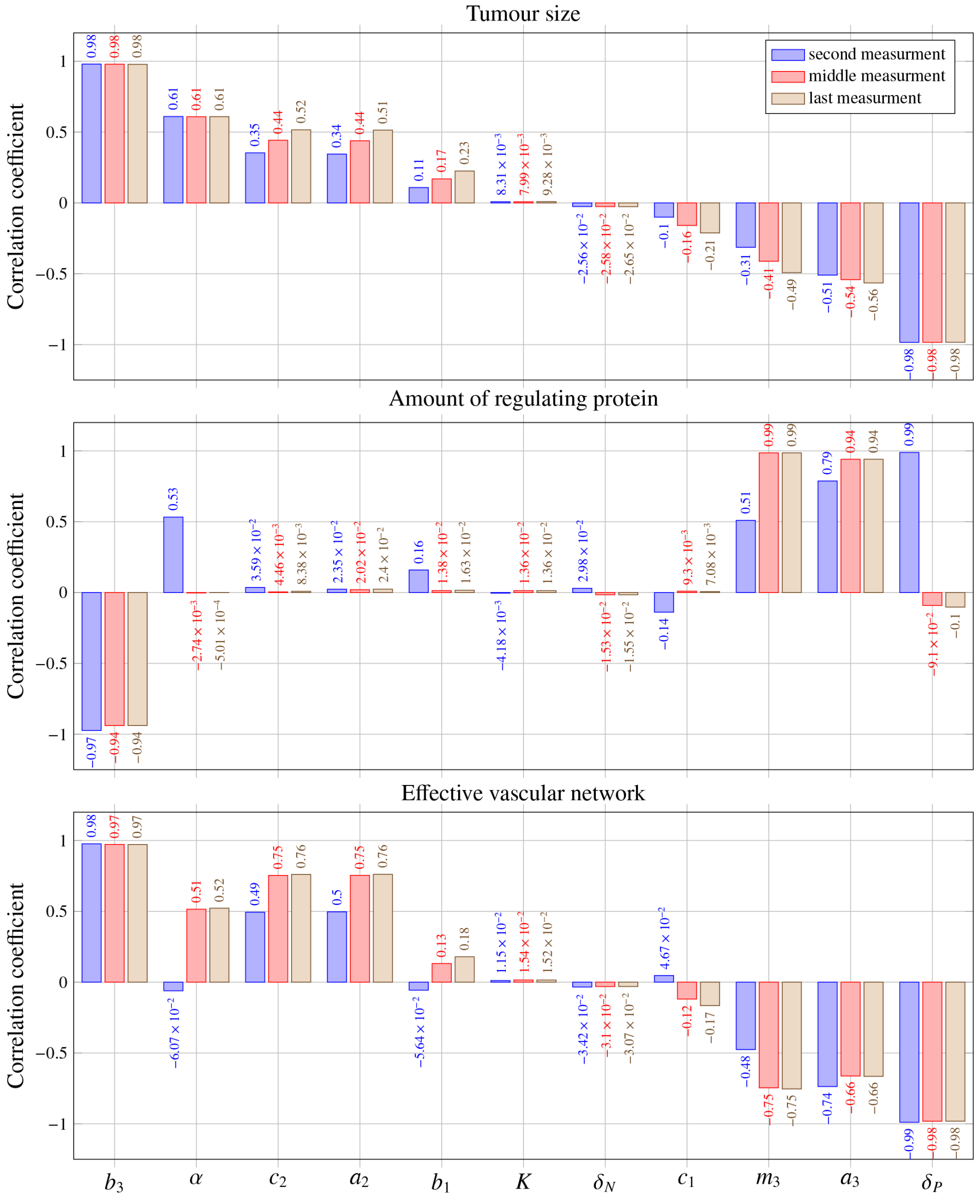

Figure S2.1. Tornado plots of partial rank correlation coefficients. Plots show the importance of each model parameter's uncertainty in the number of tumour cells (N), proteins (P) and effective vessel network (E) for three different time points corresponding to the second measurement (blue), the sixth measurement (red), and the last measurement (brown) in the experiment described in [38]. Corresponding to times $t \approx 3$, $t \approx 23$, and $t \approx 43$, respectively. For all parameters uniform probability distributions on ±2% uncertainty intervals were used according to the estimated values given in Table 3. The particular values of the correlation coefficients are given below/above each bar.

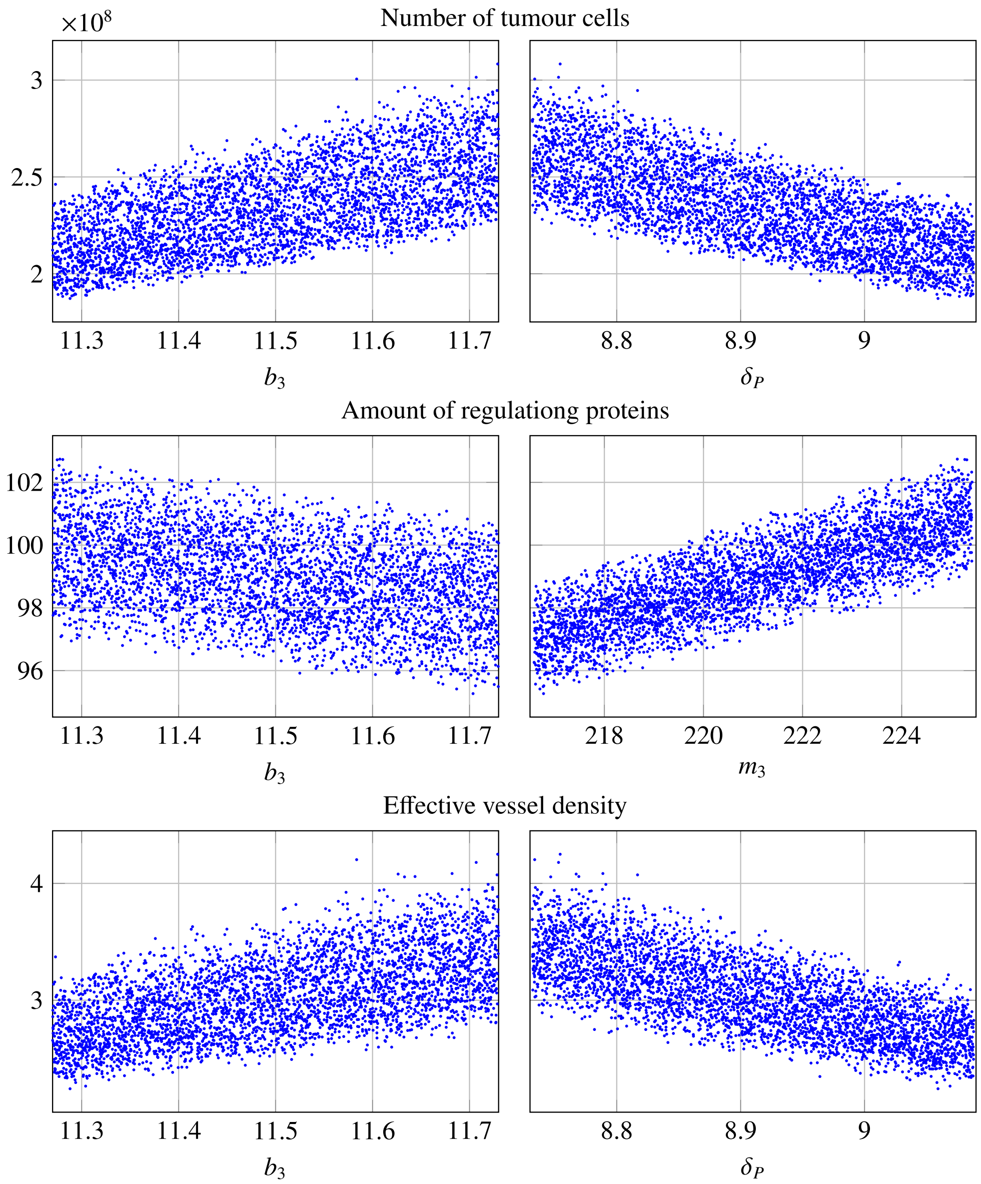

Figure S2.2. Scatter plots presenting the correlation between the value of the solution and for the most sensitive model parameters. For all parameters uniform probability distributions on ±2% uncertainty intervals were used according to the estimated values given in Table 3.

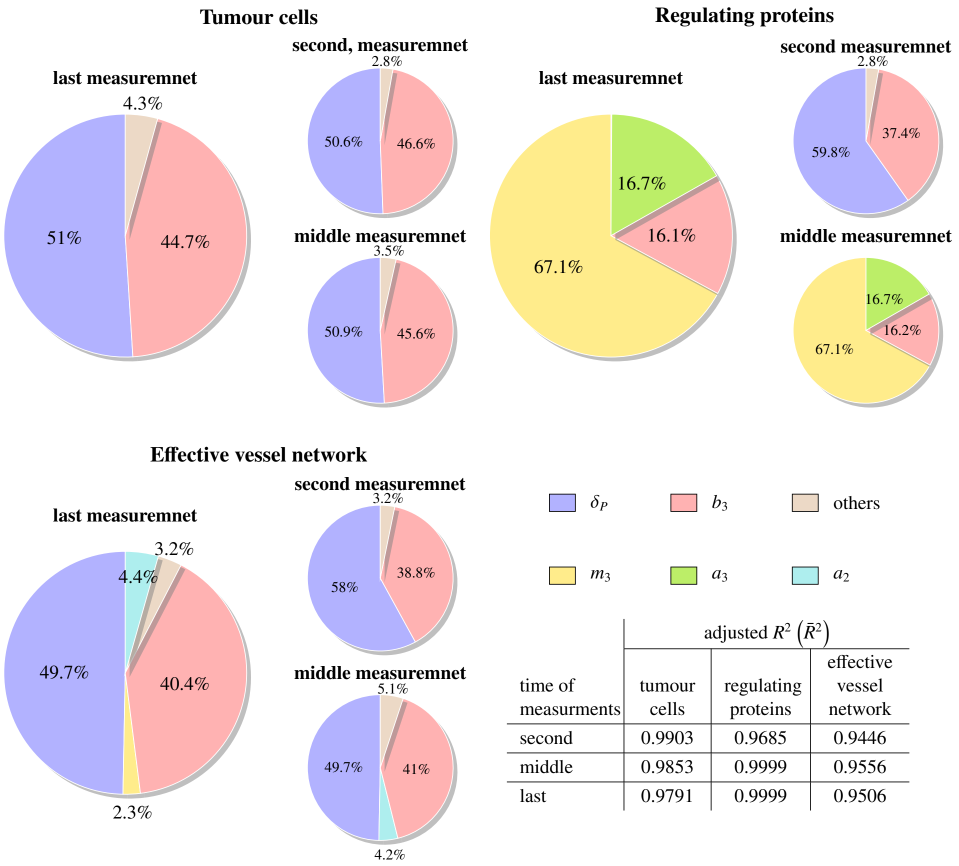

time of measurments	adjusted R^2 $\left(\bar{R}^2\right)$ tumour cells	regulating proteins	effective vessel network
second	0.9903	0.9685	0.9446
middle	0.9853	0.9999	0.9556
last	0.9791	0.9999	0.9506

Figure S2.3. Factor of prioritisation sensitivity indexes. Pie charts show the dominance (given in percentage) of particular model parameters (indicated by the same colour for each case except the ones indicates as *others*) for the same target outputs of Fig. S2.1.
